# Compositional and Hollow Engineering of Silicon Carbide/Carbon Microspheres as High-Performance Microwave Absorbing Materials with Good Environmental Tolerance

**DOI:** 10.1007/s40820-024-01369-6

**Published:** 2024-04-02

**Authors:** Lixue Gai, Yahui Wang, Pan Wan, Shuping Yu, Yongzheng Chen, Xijiang Han, Ping Xu, Yunchen Du

**Affiliations:** 1 MIIT Key Laboratory of Critical Materials Technology for New Energy Conversion and Storage, School of Chemistry and Chemical Engineering, Harbin Institute of Technology, Harbin, 150001 People’s Republic of China; 2 Anhui Provincial Laboratory of Advanced Laser Technology, College of Electronic Engineering, National University of Defense Technology, Hefei, 230037 People’s Republic of China

**Keywords:** SiC/C composites, Compositional engineering, Hollow engineering, Microwave absorption, Environmental tolerance

## Abstract

Microwave absorbing materials (MAMs) characterized by high absorption efficiency and good environmental tolerance are highly desirable in practical applications. Both silicon carbide and carbon are considered as stable MAMs under some rigorous conditions, while their composites still fail to produce satisfactory microwave absorption performance regardless of the improvements as compared with the individuals. Herein, we have successfully implemented compositional and structural engineering to fabricate hollow SiC/C microspheres with controllable composition. The simultaneous modulation on dielectric properties and impedance matching can be easily achieved as the change in the composition of these composites. The formation of hollow structure not only favors lightweight feature, but also generates considerable contribution to microwave attenuation capacity. With the synergistic effect of composition and structure, the optimized SiC/C composite exhibits excellent performance, whose the strongest reflection loss intensity and broadest effective absorption reach − 60.8 dB and 5.1 GHz, respectively, and its microwave absorption properties are actually superior to those of most SiC/C composites in previous studies. In addition, the stability tests of microwave absorption capacity after exposure to harsh conditions and Radar Cross Section simulation data demonstrate that hollow SiC/C microspheres from compositional and structural optimization have a bright prospect in practical applications.

**Supplementary Information:**

The online version contains supplementary material available at 10.1007/s40820-024-01369-6.

## Highlights

- Hollow SiC/C microspheres with controllable composition have been successfully synthesized by simultaneously implementing compositional and structural engineering.
- The optimum dielectric properties (i.e., conductivity loss and polarization loss) and impedance matching characteristics can achieve outstanding microwave absorption performance.
- Broadband wave absorption (5.1 GHz with only 1.8 mm thickness), high efficiency loss (− 60.8 dB at 10.4 GHz) combined with good environmental tolerance, demonstrate their bright prospects in practice.

**Supplementary Information:**

The online version contains supplementary material available at 10.1007/s40820-024-01369-6.

## Graphical abstract

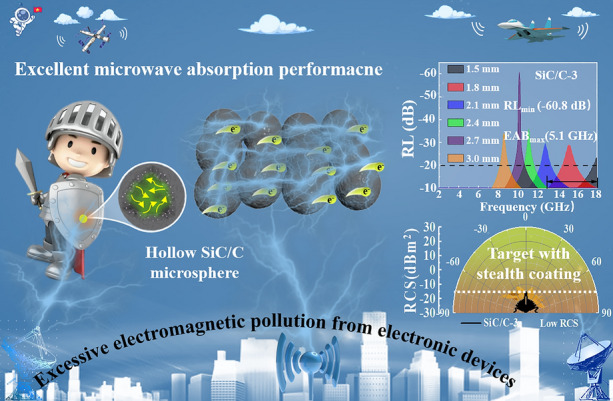

## Introduction

The advancement of electronic technology has ushered in the intelligent information era. Humans are now enjoying the benefits of technological development more than ever before in history [1–3]. However, we also face the challenge of spatial electromagnetic (EM) contamination caused by the widespread use of communication equipment, which has become a Gordian knot in both civil and military fields [4, 5]. It is therefore essential to take stringent measures to combat this situation. Conventionally, microwave absorbing materials (MAMs) have been widely recognized as the most promising functional materials for converting ambient EM waves into Joule heat through dielectric loss, magnetic loss, and interference phase cancellation, thereby suppressing or eliminating the gradually expanding EM pollution [6, 7]. To date, many researches have been endeavored to constructing MAMs with compatible magnetic and dielectric components to achieve good impedance matching and strong EM attenuation capability simultaneously [8, 9]. In particular, some magnetic metal/carbon composites (e.g., Fe/C, Co/C, Ni/C, and FeCo/C) have made great strides forward in terms of reflection loss (RL) value reduction and response bandwidth extension [10–13]. However, in addition to the critical performance of MAMs, their environmental tolerance is also an extremely important assessment indicator under some rigorous conditions, because it determines the service duration of MAMs in practical application [14]. Although magnetic metals/carbon composites can produce good microwave absorption performance, the inherent imperfections of magnetic metals, including high density and susceptibility to corrosion/oxidation, also make them difficult to provide long-time service under some rigorous natural environments, such as sun exposure, acid rain, and seawater [3, 15]. Therefore, it is of great realistic significance to develop advanced MAMs that integrate the merits of powerful microwave absorption performance and good environmental tolerance.

Silicon carbide (SiC), with diverse microscopic morphology and abundant polymorphs, is a type of very important functional nanomaterials, which have become good catalysts, semiconductors, functional ceramics, and high-frequency electronics due to their outstanding physical, chemical, electrical, and optical properties [16]. In particular, their relatively low density, excellent thermal stability, and acid/alkali resistance properties give them clear advantages as MAMs with good environmental tolerance [17]. However, the relatively wide band gap of SiC results in slow electron migration and consequently weak dielectric properties, hindering its widespread application in the field of microwave absorption [18]. Compared with SiC, carbon materials not only display tailorable dielectric loss ability, but also have good chemical stability, diversified microstructure/morphology, and abundant source, as well as broad compatibility with other EM components [19]. Previous literature has clearly demonstrated that the combination of SiC and carbon materials is an effective way to improve the dielectric properties of final composites [20]. For example, Huang et al. planted CNTs on SiC fibers through chemical vapor deposition, and they found that the formation of CNTs could greatly promote the dielectric loss capability and microwave absorption performance of SiC fibers [21]. However, commercial SiC particles or fibers usually have large size, and thus a routine post-treatment method cannot ensure the enough interaction between SiC and carbon components, which means that there still is room to consolidate their microwave absorption performance. As an alternative method, polymer-derived ceramics (PDCs) process is widely employed to prepare homogeneous SiC/C composites through the pyrolysis of polycarbosilane (PCS) [17]. Although SiC nanoparticles can be in situ generated and uniformly dispersed in carbon matrix with this method, the extremely high pyrolysis temperature of PCS brings considerable difficulties in compositional optimization and EM reinforcement. This situation suggests that a straightforward strategy for the fabrication of SiC/C composites with controllable composition and good chemical homogeneity is urgently needed and highly desirable.

Apart from the importance of composition optimization and component distribution, structural engineering is receiving more and more attention in the design of high-performance MAMs, because a profitable microstructure not only favors impedance matching, but also promotes the energy consumption of incident EM wave through its multiple reflection behavior [22]. In the past few years, hollow microsphere is always a popular and advanced structure for MAMs due to its superiorities in low density, strong attenuation ability, and good dispersion [23]. Several groups therein show keen interests in the fabrication of hollow SiC/C microspheres to further strengthen their microwave absorption characteristics [7, 20, 24]. A universal preparative strategy for such composites is to employ SiO_2_ microspheres as both Si source and hard template, and then remove excessive SiO_2_ cores with hydrofluoric acid after solid reaction with carbon shells at extremely high temperature (about 1400 °C). Although current successful examples all validate the contribution from hollow structure, it has to be pointed that this strategy usually requires strict reaction conditions, because the hollow structure easily suffers from fragmentation and collapse due to the susceptible to high-temperature solid reaction [24]. As a result, it is still seldom explored to simultaneously implement compositional and structural engineering for the construction of hollow SiC/C microspheres.

Herein, for the first time, we successfully prepare hollow SiC/C microspheres through a heterogeneous interfacial anti-interaction strategy, where phenolic resin (PR) microsphere and SiO_2_ layer are selected as the core and the shell of the precursor, respectively. The in situ generated SiC nanoparticles are uniformly dispersed in carbon shells, and their content can be rationally regulated from 27.7 to 39.4%. With the dual supports from composition and structure engineering, hollow SiC/C microspheres exhibit excellent microwave absorption performance in terms of both broadband absorption (5.1 GHz) and strong RL (− 60.8 dB), surpassing the performance of many previously reported SiC/C composites. More importantly, the stability testing of microwave absorption performance after exposure to rigorous conditions and Radar Cross Section (RCS) simulation demonstrate that SiC/C composites have good environmental tolerance and excellent radar stealth performance in practical applications.

## Experimental Section

### Materials

All chemicals, including absolute ethanol (EtOH), cetyltrimethyl ammonium bromide (CTAB, 99%), ammonium hydroxide solution (NH_3_·H_2_O, 25–28%), resorcinol (C_6_H_6_O_2_, 99%), formaldehyde (CH_2_O, 37 wt%), tetraethyl orthosilicate (TEOS, > 99%), magnesium powder (Mg, > 99%), were purchased from Aladdin Technology Co. Ltd., Shanghai, China. The above chemicals were of analytical grade and were used directly, and deionized (DI) water was produced by an OKP-S040 standard ultrapure water system and used in all experiments.

### Synthesis of Core–Shell PR@SiO_2_/PR Microspheres

In a typical synthesis, 1 g of CTAB was dissolved in a solution consisting of 50 mL of DI water, 20 mL of EtOH and 0.3 mL of NH_3_·H_2_O, and stirred vigorously for 10 min at room temperature. Then, 1.28 g of resorcinol and 0.74 mL of formaldehyde were added separately to the above solution to form a milky solution, after which a certain amount of TEOS was injected and the mixed solution was stirred continuously for a further 1 h. Finally, the mixture was sealed in the 100 mL Teflon-lined autoclave and maintained at 100 °C for 24 h. After the reaction was naturally cooled to room temperature, the solution was centrifuged to get brown product, which was further washed three times with ethanol and DI water and dried at 60 °C for 12 h. For convenience, the TEOS/resorcinol molar ratios of 0.388, 0.582, 0.776, and 0.970 were labeled as PR@SiO_2_/PR-1, PR@SiO_2_/PR-2, PR@SiO_2_/PR-3, and PR@SiO_2_/PR-4, respectively.

### Synthesis of Hollow SiC/C Microspheres

The as-prepared core–shell PR@SiO_2_/PR microspheres were first put into porcelain boat and directly pyrolyzed under N_2_ atmosphere at 800 °C (heating rate is 5 °C min^−1^) for 3 h in a tubular furnace and cooled to room temperature to obtain the hollow SiO_2_/C-X (X = 1, 2, 3, and 4, corresponding to their PR@SiO_2_/PR-X precursors, respectively.) microspheres. Subsequently, the required amount of SiO_2_/C and magnesium powder (the mass ratio of SiO_2_/C to magnesium powder was fixed at 1:4) was added into an agate mortar, and the mixture was sufficiently ground for 10 min. Finally, the obtained samples were pyrolyzed again under N_2_ atmosphere with the different heat treatment condition for 6 h to produce the hollow SiC/C microspheres. For convenience, the final composites were referred to as SiC/C-X (X = 1, 2, 3, and 4, corresponding to their SiO_2_/C-X precursors, respectively). In addition, for the product with different temperature conditions, using the SiC/C-3 as an example, the SiC/C-3 of 700, 800, and 900 °C were labeled as SiC/C-3-700, SiC/C-3-800, and SiC/C-3-900, respectively. To ensure that pure SiC/C composites were obtained, the above pyrolysis products were placed in hydrochloric acid (HCl, 3 mol L^−1^) for over 6 h to remove the remaining impurity phases MgO. The other details of this work including materials characterization, electromagnetic parameter measurement, and computer simulation technology was available from the supporting information in the Springer Online Library.

## Results and Discussion

### Preparation and Structure Characterizations of SiC/C Composites

**Fig. 1:**
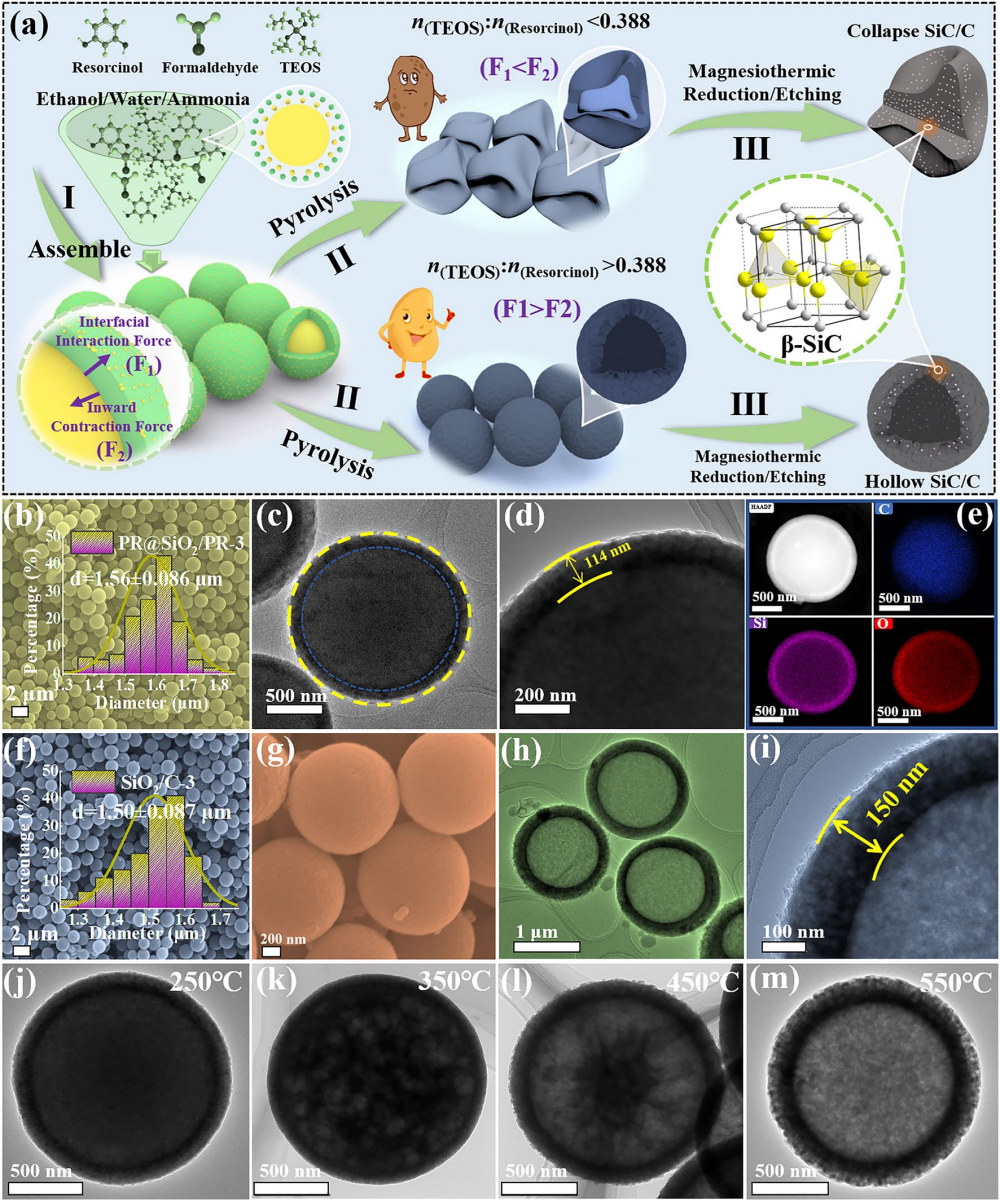
**a** Preparation mechanism diagram of SiC/C composites. **b** SEM images, **c-d** TEM images and **e** the corresponding element mapping images of PR@SiO_2_/PR-3. **f-g** SEM images and **h-i** TEM images of SiO_2_/C-3. **j-m** TEM images with different pyrolysis temperature (250, 350, 450, and 550 °C) of SiO_2_/C-3

Figure 1a illustrates the step-by-step preparation procedures of SiC/C composites with a heterogeneous interface anti-contraction strategy. First, resorcinol and formaldehyde are sequentially dispersed in alkaline aqueous ethanol solution to generate phenolic resin (PR) microspheres. After the introduction of TEOS, silica oligomers from the hydrolysis will be co-assembled with the residual PR oligomers to produce core–shell PR@SiO_2_/PR microspheres. Scanning electron microscopy (SEM) images show that all the resultant PR@SiO_2_/PR microspheres have regular spherical morphology, good dispersion, and smooth surface, and their average diameters range from 1.27 ± 0.079 to 1.61 ± 0.086 μm with increasing the dosage of TEOS (Figs. 1b and S1). Transmission electron microscopy (TEM) images identify the core–shell configuration in PR@SiO_2_/PR-3, as well as the thickness of the external shells at about 114 nm (Fig. 1c, d). EDS mapping results further firmly support that a desirable core–shell configuration has been successfully created in these microspheres, because O and Si elements are mostly distributed in an outer ring, whose size is obviously larger than the distribution region of C element (Fig. 1e), and meanwhile, a fraction of C atoms can be also observed in the region of O and Si elements, implying the presence of PR in theshells. It is found that the dosage of TEOS plays an important role in maintaining the spherical morphology of these composites during high-temperature pyrolysis. For example, SiO_2_/C-1 derived from PR@SiO_2_/PR-1 (the TEOS/resorcinol molar ratio of 0.388) is composed of numerous wizened particles and almost completely loses its original morphology (Fig. S2a, b), and in contrast, the other intermediate composites with higher TEOS dosages (the molar ratio of TEOS/resorcinol is more than 0.582), i.e., SiO_2_/C-2, SiO_2_/C-3, and SiO_2_/C-4, all inherit the spherical morphology very well except a slight shrinkage in the average diameter (Figs. 1f, g and S2c, d). Of note is that some impurities are detected in SiO_2_/C-4, suggesting that the dosage of TEOS in this case may be a little excessive. TEM characterization is further carried out by taking SiO_2_/C-3 as a representative sample. Compared with its precursor (PR@SiO_2_/PR-3), SiO_2_/C-3 not only displays an unexpected hollow structure, but also gives a thicker shell at about 150 nm (Fig. 1h, i). According to previous studies, when some pure organic precursors, e.g., polymers and MOFs, are pyrolyzed under high-temperature inert atmosphere, there will be a dramatic inward shrinkage behavior, resulting in the formation of carbon-based products with much smaller size [25, 26]. However, if a stable external shell is pre-constructed on the surface of organic precursors, the interaction between the shell and the precursor will induce preferential carbonization at the interface and produce an interfacial interaction force (F1) to resist the inward contraction force (F2), and more importantly, such an interfacial interaction will also promote the inside-out diffusion of organic precursors and finally create an interior cavity [27]. The microstructure evolution from PR@SiO_2_/PR-3 to SiO_2_/C-3 at different pyrolysis temperature indeed records the process of gradual internal cavitation (Figs. 1j–m and S3). When the pyrolysis temperature is 250 °C, the intrinsic microstructure remains consistent with the precursors, indicating that the contraction process of the heterogeneous interface between the core and the shell has not been triggered yet. As the temperature continues to increase to 350 °C, it is observed that the heterogeneous interfaces boundaries between the core and the shell are not as dense as they are at first, and their interior regions have many apparent holes with different size. With a further increase of the temperature to 450 °C, the PR core undergoes continuous decomposition, resulting in the gradual aggregation of small pores into some larger ones. Upon reaching a temperature of 550 °C, the internal hollow structure is completely formed, and the spherical morphology can still be maintained without obvious collapse and fragmentation phenomenon. These results indicate that the formation of hollow SiO_2_/C composites follows the mechanism of heterogeneous interface anti-contraction and also explains why the thickness of the external shells increases. The collapse of SiO_2_/C-1 can be attributed to the fact that the relatively thin SiO_2_ shells fail to provide enough interfacial interaction force (Fig. 1a).

The intermediate SiO_2_/C composites are further converted into corresponding SiC/C microspheres by magnesiothermal reduction, and the reaction mechanism can be explained by the following reaction equation [28]:

1 $${\text{SiO}}_{2} \left( {\text{s}} \right) + 2{\text{Mg}}\left( {\text{g}} \right) + {\text{C}}\left( {\text{s}} \right) \to {\text{SiC}}\left( {\text{s}} \right) + 2{\text{MgO}}\left( {\text{s}} \right)$$

**Fig. 2:**
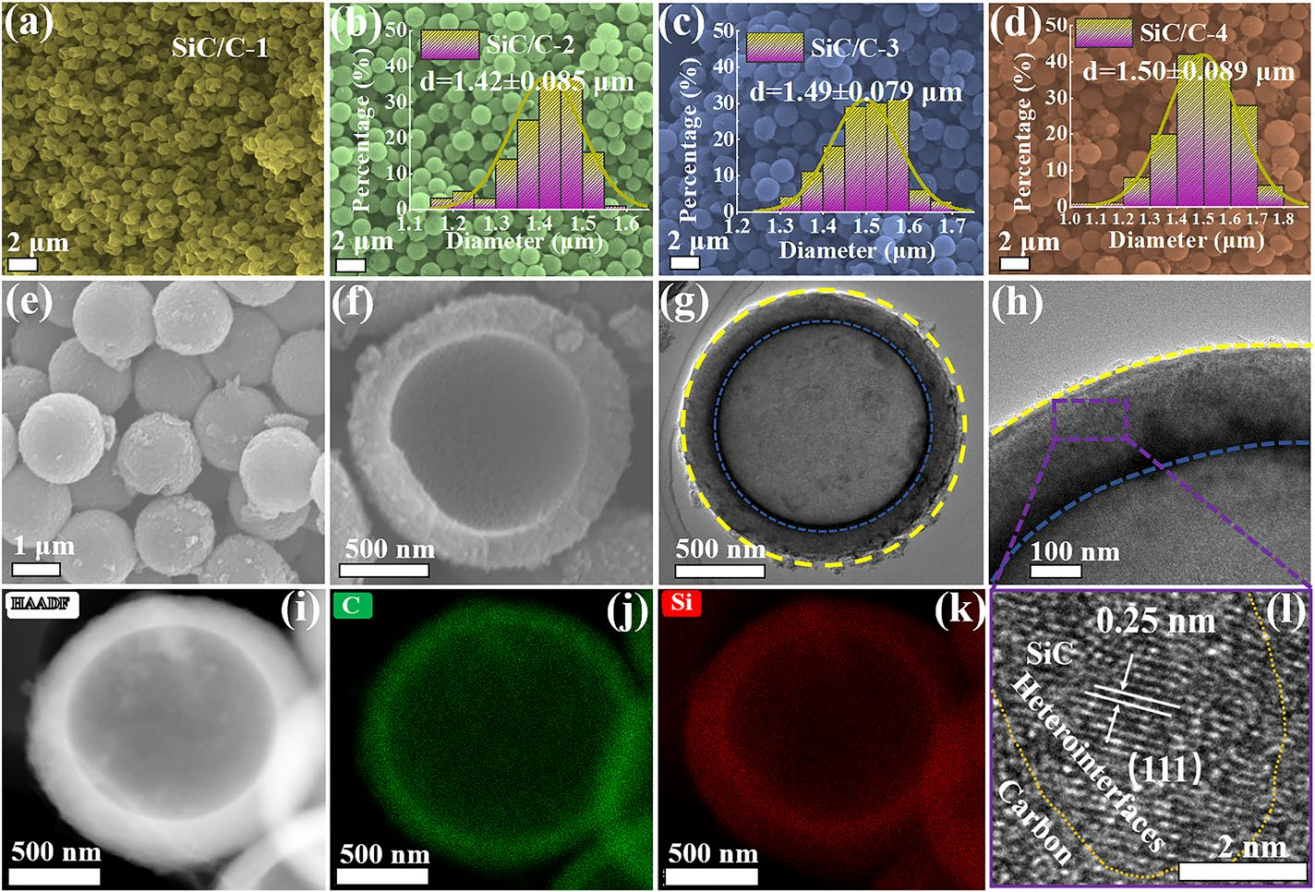
Low-magnification SEM images of **a** SiC/C-1, **b** SiC/C-2, **c** SiC/C-3, and **d** SiC/C-4, and the insets are the corresponding statistical distribution of diameters. **e–f** High-magnification SEM images of SiC/C-3. **g–h** TEM images of SiC/C-3. **I–k** The corresponding element mapping images and **l** HR-TEM image of SiC/C-3

Thanks to the good chemical homogeneity of SiO_2_/C composites, SiO_2_ and carbon species have full contact (Fig. 1c), which effectively ensures the generation of crystalline SiC nanoparticles. The by-product, MgO, can be easily removed by HCl treatment. It is very interesting that all composites exhibit very similar morphologies to those of SiO_2_/C precursors, and their statistical distribution of diameters also shows no significant change, suggesting that the carbon shells in these microspheres are enough stable to survive from the intensive magnesiothermal reaction (Fig. 2b–d). Both SEM image on a broken microsphere and TEM images elucidate that hollow structure has been well inherited from SiO_2_/C composites, even after the magnesiothermal reaction (Fig. 2e–h). HR-TEM image identifies some ultrafine SiC nanoparticles in carbon shells, and their size is less than 10 nm (Fig. S4a). The formation of SiC nanoparticles is inevitably involves the nucleation and growth processes, as well as the diffusion of carbon and silicon species. Carbon shells are hard carbon derived from phenolic resin, and thus they will slow down the diffusion of silicon species significantly and further affect the nucleation and growth processes. In other words, carbon shells provide effective space confinement effect, responsible for the very small size of SiC nanoparticles. The lattice spacing of 0.25 nm corresponds to the (111) plane of *β*-SiC with a typical cubic structure (Fig. 2l). Again, the elemental mapping results confirm the homogeneous distribution in final SiC/C composites (Fig. 2i–k). We also attempt to determine the density of SiC/C-3 by measuring the changes in mass and volume of the mixture of SiC/C-3 and wax. The result shows the density of SiC/C-3 is only 1.7 g cm^−3^, which is obviously smaller than those of commercial carbon power (2.0 g cm^−3^) and silicon carbide powder (2.5 g cm^−3^), implying that hollow structure endows SiC/C-3 with lightweight feature.

**Fig. 3:**
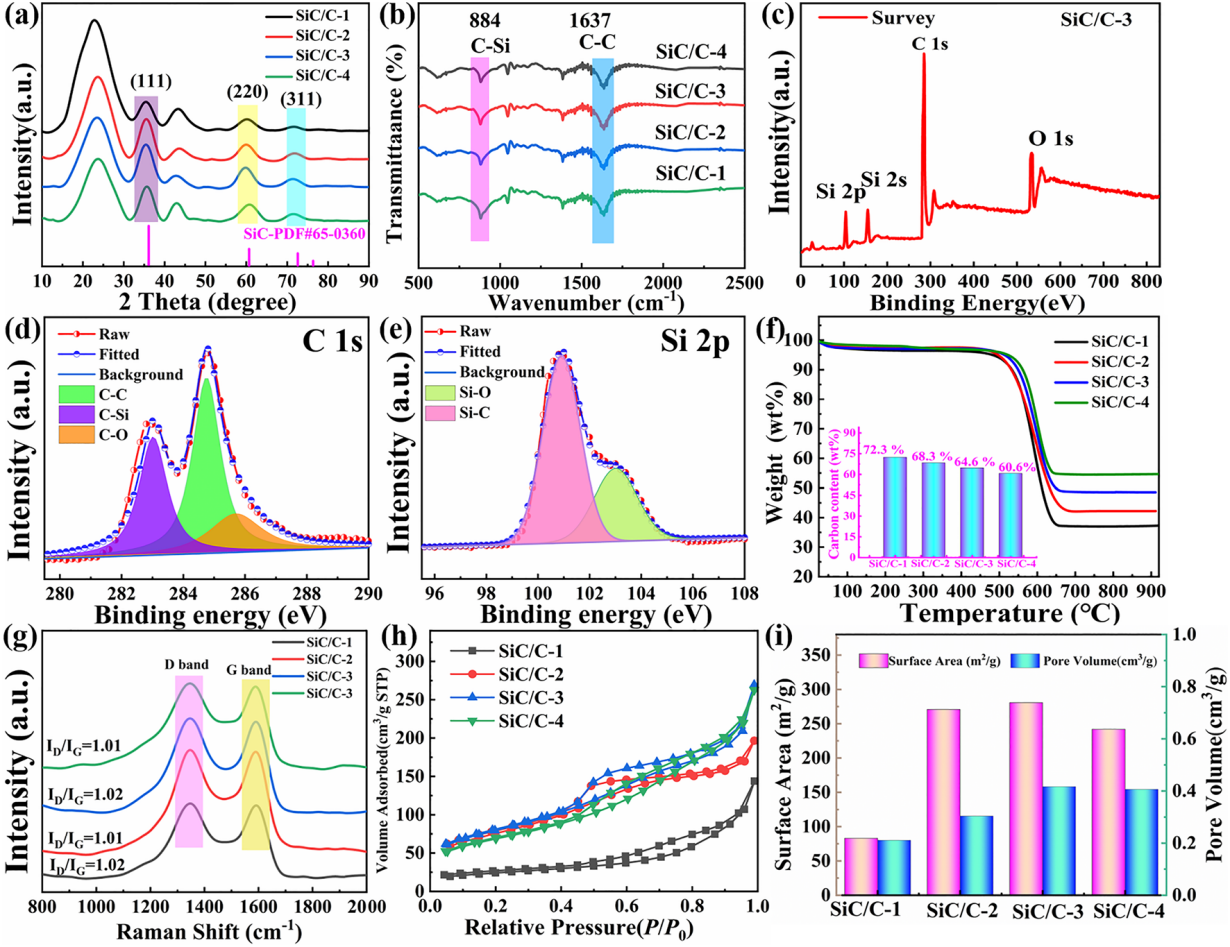
**a** XRD patterns, **b** FT-IR spectra, **c-e** XPS survey spectra, and **f** TG curves (Inset is the calculated contents of carbon), **g** Raman spectra, **h** N_2_ adsorption–desorption isotherms, and **i** the histograms of BET surface areas and pore volumes of SiC/C composites

The crystallographic structure and phase evolution during the preparative process are also studied by X-ray diffraction (XRD, Figs. 3a and S4b). Both PR@SiO_2_/PR and SiO_2_/C exhibit a broad peak at about 23.2° that is typically associated with amorphous species [8], suggesting that carbon shells from the first step of pyrolysis are still amorphous overall. However, SiO_2_/C also gives an additional small peak at approximately 44.0°, which is usually considered to be related to the formation of some tiny crystalline domains inside carbon shells [29]. For SiC/C composites, three new diffraction peaks at 35.4°, 60.3°, and 71.9° indexed to the (111), (220), and (311) planes of* β*-SiC (PDF#65–0360) can be clearly detected, which manifests that the magnesiothermal reaction indeed induces the generation of SiC nanoparticles. With Scherrer’s equation, the average sizes of SiC nanoparticles in these composites are all close to 5 nm, in good agreement with TEM results (Fig. S4a). Of note is that the peaks at 23.2° and 44.0° still in exist in final SiC/C composites. Such a situation means that carbon shells are less impacted by the magnesiothermal reaction and also explains why SiC/C-2, SiC/C-3, and SiC/C-4 can well preserve their morphologies and structures. As two sensitive tools to collect the information of chemical bonds, Fourier transform infrared (FT-IR) spectroscopy and X-ray photoelectron spectroscopy (XPS) are also employed to characterize final SiC/C composites. It can be observed that there are two typical absorption bands at 884 and 1637 cm^−1^, and they are from the stretching modes of Si–C bond and C–C bond [30], respectively, again verifying the generation of SiC nanoparticles (Fig. 3b). Compared with SiO_2_/C composite, the characteristic band for the asymmetry stretching vibration of Si–O–Si at 1100 cm^−1^ almost disappears in SiC/C composites (Fig. S4c), which implies complete conversion from SiO_2_ to SiC during magnesiothermal reaction. The characteristic peaks at 532.9, 284. 6, and 105.6 eV in the survey spectrum of XPS can be ascribed to O 1* s*, C 1* s* and Si 2*p*, respectively (Fig. 3c). Amorphous carbon shells have rich surface functional groups and defect sites, thus facilitating the involvement of O element. The deconvolution results of C 1* s* show the distinct peaks of C-Si (282.9 eV), C–C (284.6 eV), and C-O (285.7 eV) bonds (Fig. 3d) [30], and the corresponding Si 2*p* not only confirms the dominance of Si–C bond (Fig. 3e) [31], but also reveals the partial oxidation of SiC nanoparticles on the surface of SiC/C composites.

The content and relative graphitization degree of carbon species in carbon-based composites are always considered as two crucial factors that can greatly affect their dielectric properties [32]. Figure 3f shows thermogravimetric (TG) curves of different SiC/C composites under air atmosphere, and all samples exhibit very similar profiles that contain a slight weight decrease (less than 5%) between 25 and 490 °C, as well as an intensive weight loss in the temperature range of 490–650 °C. The slight weight decrease is reasonably attributed to the removal of physically absorbed water and surface functional groups, and the intensive weight loss is caused by the combustion of the carbonaceous components. Although SiC is generally stable under high-temperature air atmosphere, SiC nanoparticles in these composites has very small size (less than 10 nm), and thus they will also be oxidized when the temperature reaches 900 °C. Actually, we treat the composite of SiC/C-3 at 900 °C for 0.5 h in a muffle furnace, and finally harvest white powder without any characteristic peaks of SiC (Fig. S4d). By considering the negligible weight change in the temperature spans before 490 and after 650 °C, the oxidation of SiC nanoparticles is highly likely to occur synchronously with the combustion of the carbonaceous components. Based on the specific percentages of the residues after TG test, and the specific carbon contents in SiC/C composites can be calculated by Eq. (2):

2 $${\text{wt\% carbon}} = 1 - {\text{wt\% water}} - {\text{wt\% }}R\frac{{M\left( {{\text{SiC}}} \right)}}{{M\left( {{\text{SiO}}_{2} } \right)}}$$

where wt% water and wt% *R* represent the weight percentage of trace absorbed water and the weight percentage of the residue after combustion, and *M*(SiC) and *M*(SiO_2_) refer to the molecular weights of SiC and SiO_2_, respectively. Thus, the theoretical carbon content can be deduced as 72.3%, 68.3%, 64.6%, and 60.6% for SiC/C-1, SiC/C-2, SiC/C-3, and SiC/C-4, respectively. All the above results definitely support that compositional and hollow engineering have been successfully applied to final SiC/C composites. Raman spectra are employed to disclose the difference in the bonding state of carbon atoms, i.e., relative graphitization degree, of carbon components in composites [33]. As observed, all SiC/C composites display two bands at ~ 1350 and ~ 1580 cm^−1^, corresponding to D band active in disordered arrangement of carbon atoms and G band only generated at *sp*^2^ sites, respectively. It is very interesting that these four composites give pretty close curve profiles and *I*_D_/*I*_G_ (the intensity ratio of D band to G band) values (Fig. 3g). This phenomenon validates that carbon components in these composites have quite similar relative graphitization degree, and thus the changeable composition and structure will be taken as the primary reasons responsible for their different dielectric properties. Figure 3h presents adsorption/desorption isotherms of different SiC/C composites. Although all of them exhibit IV-type isotherms according to the classification of the International Union of Pure and Applied Chemistry, the N_2_ uptake of SiC/C-1 is obviously smaller than those of other composites. Apparently, the structure collapse leads to the decrease of porosity in SiC/C-1. As a result, its specific surface area (*S*_BET_) and total pore volume (*V*_t_) are also less than those SiC/C-2, SiC/C-3, and SiC/C-4 (Fig. 3i). In addition, the pore size distributions of SiC/C composites are provided in Fig. S5. One can see that the change in the pore size distribution does not present a regular trend, while the most probable distributions of these composites are all centered between 3 and 10 nm. This is because these mesopores are generated by the etching of unreacted SiO_2_ as well as the release of carbon-containing small molecules during the pyrolysis process. It is worth noting that the size of these mesopores is several orders of magnitude lower than the wavelength of incident EM wave, and thus they will not induce multiple reflection behaviors EM wave, but according to Maxwell-Garnet theory, the presence of mesoporous structure can regulate the dielectric constant of MAMs and then improve impedance matching, resulting in an indirect contribution to microwave absorption [34]. Moreover, the presence of mesoporous structure will reduce the overall density of MAMs and endow them with lightweight feature.

### Microwave Absorption Performance of SiC/C Composites

RL intensity and effective absorption bandwidth (EAB) are two important indicators to evaluate the overall microwave absorption characteristics of MAMs [35, 36], where the former represents the attenuation ability of MAMs toward incident EM wave at a given frequency and the latter describes the frequency range in which MAMs can produce RL intensity less than an appointed value (the threshold is usually set at -10.0 dB because 90% of EM energy will be dissipated in that case [37].

**Fig. 4:**
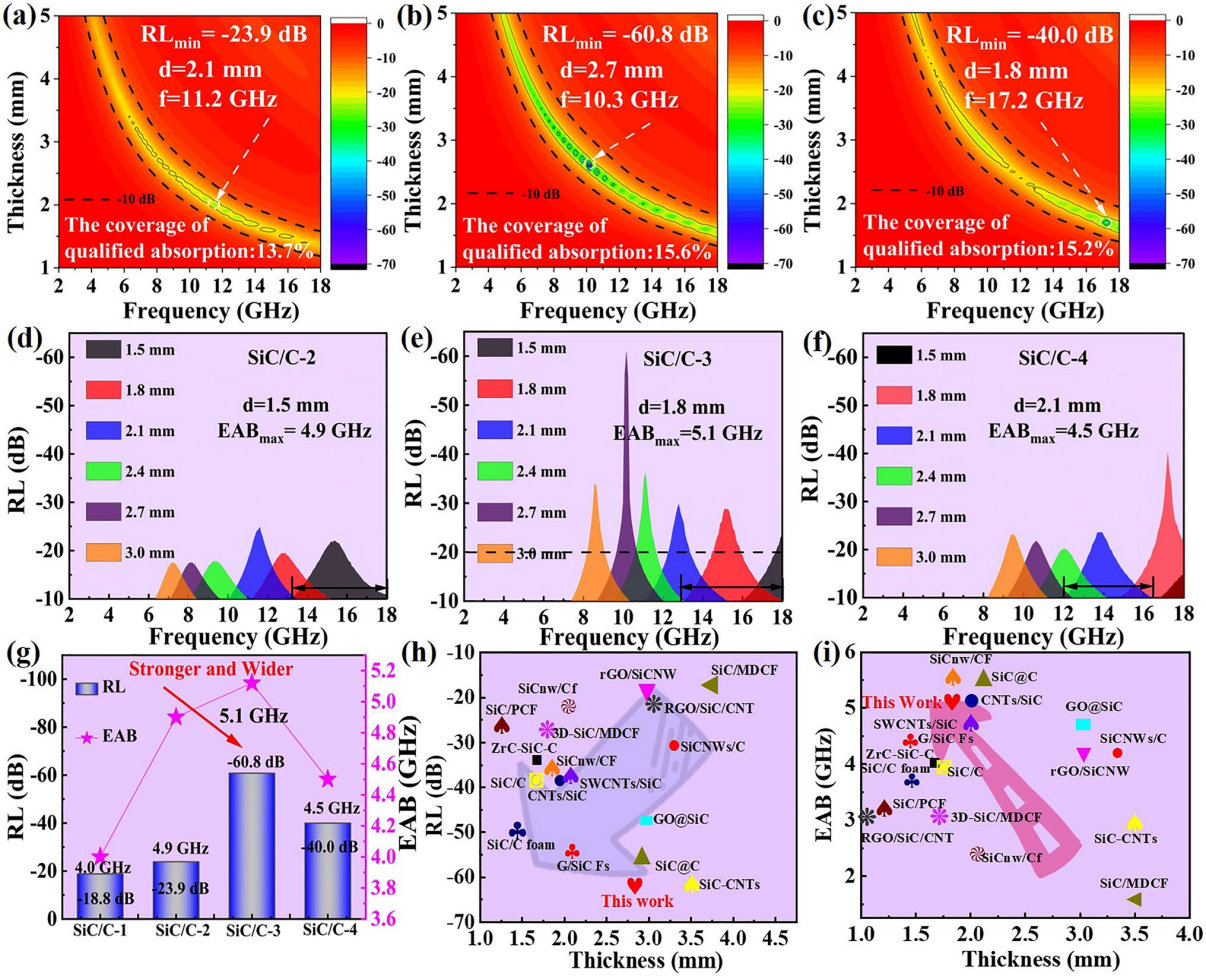
2D RL mapping of **a** SiC/C-2, **b** SiC/C-3 and **c** SiC/C-4. RL curves below -10 dB at given absorber thickness of **d** SiC/C-2, **e** SiC/C-3 and **f** SiC/C-4. **g** The frequency dependence of *RL*_min_ and EAB values of all samples with the optimal thicknesses. **h** Comparison of *RL*_min_ values and **i** EABs among previously reported silicon carbide and carbon related MAMs

Figures S6a and 4a-c display 2D projection diagrams of different SiC/C composites with frequency (2.0–18.0 GHz) and absorber thickness (1.0–5.5 mm) as two independent variables. From these diagrams, one can find that all these composites can dissipate EM energy to some extent, while their specific performance is obviously distinguishable. On one hand, they have quite different *RL*_min_. As observed, *RL*_min_ value of SiC/C-1 is only − 18.8 dB at 5.9 GHz with an absorber thickness of 5.0 mm. As the content of SiC nanoparticles is gradually increased and the hollow structure is well maintained, *RL*_min_ value of other composites will be significantly improved. For example, SiC/C-2 and SiC/C-3 harvest their *RL*_min_ values of − 23.9 dB (11.2 GHz, 2.1 mm) and − 60.8 dB (10.3 GHz, 2.7 mm), while with more SiC nanoparticles, *RL*_min_ value of SiC/C-4 falls back to − 40.0 dB (17.2 GHz, 1.8 mm). On the other hand, the coverage of qualified absorption (i.e., *RL*_min_ < -10.0 dB) is also different, and they are 13.0%, 13.7%, 15.6%, and 15.2% for SiC/C-1, SiC/C-2, SiC/C-3, and SiC/C-4, respectively. For a more intuitive comparison in EABs, we further plot RL curves with some given absorber thickness (e.g., 1.5, 1.8, 2.1, 2.4, 2.7, and 3.0 mm) in Figs. S6b and 4d-f. It seems that the broadest EAB has a similar trend to that of *RL*_min_ intensity. SiC/C-1 achieves its best EAB in the frequency range of 13.1–17.1 GHz with an absorber thickness of 2.1 mm. Both SiC/C-2 (4.9 GHz) and SiC/C-3 (5.1 GHz) generates wider EABs than SiC/C-1, and unfortunately, SiC/C-4 again presents an undesirable degradation in EAB (4.5 GHz). When we compare the integrated performance of these composites (Fig. 4g), it will be easy to determine that SiC/C-3 is the best candidate among these composites because it have both strong absorption and broad response.

Actually, even if we put the threshold forward to -20.0 dB corresponding to 99% of absorption intensity (Fig. 4e), it can still satisfy this high demand in the frequency ranges of 7.3–18 GHz (thickness < 3 mm). The superior microwave absorption performance of SiC/C-3 can be further validated by comparing its performance with those reported SiC/C composites in recent years (Fig. 4h, i and Table S1) [7, 20, 38–52]. It is apparent that both *RL*_min_ and EAB of SiC/C-3 are in the top level as compared with these counterparts, which firmly demonstrates the significance of compositional and hollow engineering.

### Microwave Absorption Mechanism of SiC/C Composites

**Fig. 5:**
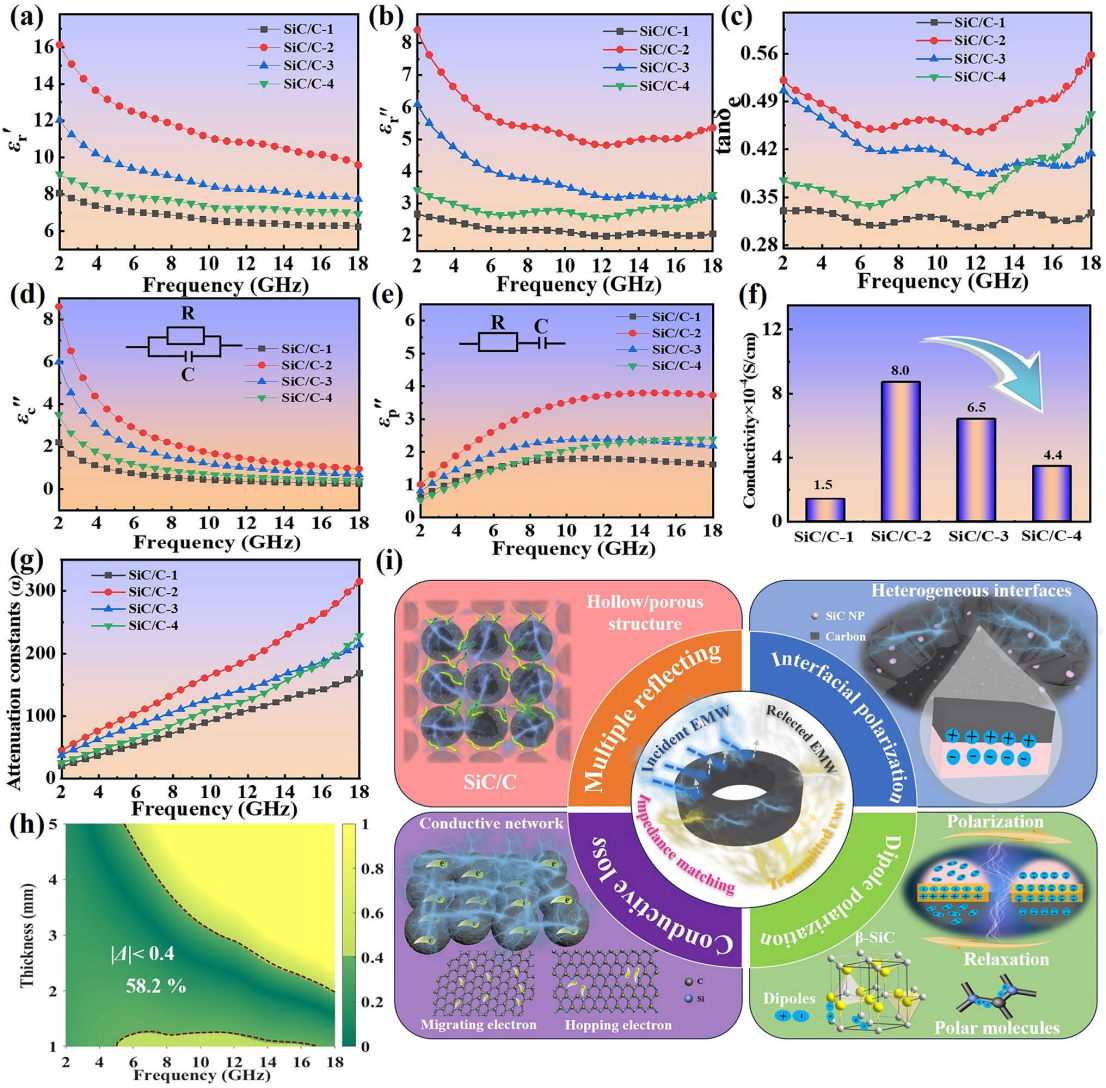
Frequency-dependent **a** *ε*_*r*_*'* curves, **b** *ε*_*r*_*"* curves, **c** dielectric loss tangent (tan*δ*e), **d** fitted conductivity loss, **e** fitted polarization loss, **f** conductivity values, **g** *α* curves of SiC/C composites. **h** 2D |*Δ*| map and **i** schematic illustration for the microwave absorption mechanisms of SiC/C-3

Given that microwave absorption characteristics are mainly determined by relative complex permittivity (*ε*_r_ = *ε*_r_' − j*ε*_r_") and complex permeability (*μ*_r_ = *μ*_r_' − j*μ*_r_") according to the transmission line theory [53, 54], and therefore, the associated EM parameters in the frequency range of 2–18 GHz are analyzed to explore the intrinsic reasons for the differences in the microwave absorption performance of SiC/C composites. As there are no any magnetic components in these SiC/C composites, their real parts and imaginary parts of relative complex permeability are almost constant and very close to 1 and 0 (Fig. S7), respectively, indicating these composites cannot dissipate EM energy through magnetic loss. Figure 5a, b shows frequency-dependent *ε*_r_' and *ε*_r_" of different SiC/C composites. Among four samples, SiC/C-1 exhibits the smallest *ε*_r_' and *ε*_r_" values, whose *ε*_r_' decreases from 8.1 at 2.0 GHz to 6.2 at 18.0 GHz and *ε*_r_" decreases from 2.5 at 2.0 GHz to 2.2 at 18.0 GHz. It is very interesting that as compared to SiC/C-1, SiC/C-2 gives much higher *ε*_r_' and *ε*_r_" values, where *ε*_r_' decreases from 16.1 at 2.0 GHz to 9.6 at 18.0 GHz and *ε*_r_" decreases from 8.4 at 2.0 GHz to 5.3 at 18.0 GHz. In general, carbon atoms in common carbon materials have two kind of different hybridization modes, i.e., *sp*^2^ and *sp*^3^, and the region with dominant *sp*^2^ carbon atoms will facilitate electron transfer due to the presence of delocalized π bond. For amorphous carbon materials, high pyrolysis temperature can increase the content of *sp*^2^ carbon sites remarkably, which means carbon materials from high temperature have good conductivity. Moreover, some heteroatoms with strong electronegativity, such as N and O, in carbon materials can also promote electron transfer, and as a result, carbon materials even exhibit better electronic conductivity than metal nanoparticles in some cases [55]. SiC, by contrast, is a typical covalent compound, whose Si and C atoms are both hybridized with *sp*^3^ mode, and thus SiC usually have very weak conductivity. Even if the presence of some heteroatoms, SiC can at most be regarded as a semiconductor. It is well documented that good conductivity is favorable for large complex permittivity [3], and thus carbon materials have larger relative complex permittivity (including *ε*_r_' and *ε*_r_") than SiC particles in our case. It is very interesting that the complex permittivity SiC/C-2 with higher SiC content overtakes that of SiC/C-1. Raman spectra have demonstrated that SiC/C-1 and SiC/C-2 possess very similar relative graphitization degree of carbon components, and thus the unexpected increases in *ε*_r_' and *ε*_r_" values should be linked with their different structures. According to Maxwell–Garnett's theory, a porous medium can be taken as an “effective medium” composed of solid phase and void phase [34, 56], and the dielectric constant of this “effective medium” ($\varepsilon_{{{\text{eff}}}}^{{{\text{MG}}}}$) may be calculated by Eq. (3):

3 $$\varepsilon_{{{\text{eff}}}}^{{{\text{MG}}}} = \varepsilon_{1} \frac{{\left( {\varepsilon_{2} + 2\varepsilon_{1} } \right) - 2v\left( {\varepsilon_{1} - \varepsilon_{2} } \right)}}{{\left( {\varepsilon_{2} + 2\varepsilon_{1} } \right) + v\left( {\varepsilon_{1} - \varepsilon_{2} } \right)}}$$

where *ε*_1_ and *ε*_2_ are the dielectric constant of solid phase and void phase, respectively, and *v* is the volume fraction of void phase. Therefore, under the condition of the same volume, a medium with high porosity is expected to produce small $\varepsilon_{{{\text{eff}}}}^{{{\text{MG}}}}$, that is, SiC/C-2 with intact hollow structure should have smaller $\varepsilon_{{{\text{eff}}}}^{{{\text{MG}}}}$ than SiC/C-1. However, in this study, the filler loading of SiC/C composites in wax matrix is calibrated by mass percentage rather than volume percentage, which means that SiC/C-2 with hollow structure will gain much higher volume fraction in wax matrix than SiC/C-1, and thus there is also a greater possibility for SiC/C-2 to create a conductive network in wax matrix and generate larger dielectric constant. Similar phenomena have been observed in some previous studies about carbon-based MAMs [57]. On the premise that the hollow structure is well maintained, the incremental content of SiC nanoparticles will moderately reduce *ε*_r_' and *ε*_r_" values. However, in high-frequency range, SiC/C-4 displays quite close *ε*_r_" values to SiC-3, which may be related to more loss contribution from interfacial polarization [58].

Figure 5c presents dielectric tangents of different SiC/C composites to evaluate their dielectric loss abilities directly, and one can find that the dielectric loss ability almost exhibits the same trend to those of *ε*_r_' and *ε*_r_" values, and only in the frequency range of 15.0–18.0 GHz, an unexpected turnover is achieved between SiC/C-3 and SiC/C-4. Dielectric loss has long been considered to come from the total contribution of conductivity loss and polarization loss [3, 59], where conductivity loss highly correlates with the transport of residual carriers in EM medium and polarization loss benefits from the thermal motion of charged particles [27]. Four-probe conductivity measurements reveal that under the same filler loading in wax (35 wt%), the conductivities of the films with SiC/C-1, SiC/C-2, SiC/C-3, and SiC/C-4 are 1.5 × 10^–4^, 8.0 × 10^–4^, 6.5 × 10^–4^, and 4.4 × 10^–4^ S cm^−1^, respectively (Fig. 5f). The change in conductivity is same as those in *ε*_r_' and *ε*_r_" values, suggesting that conductivity loss plays an important role in dielectric loss. In general, dipole orientation polarization and interfacial polarization are taken as two crucial modes that can produce significant energy consumption in the studied frequency range [25]. The former refers to the fact that the electric field causes a hysteretic reorientation process of intrinsic dipoles. This means that the change of these dipoles always lags behind the field, and they tend to acquire energy from this field to complete the rearrangement, resulting in the consumption of EM energy. The latter requires that the heterogeneous interfaces between different EM components or phases, where the difference in the space charge distribution on these interfaces will be generated. This polarization process can respond to the action of EM wave through the movement of interface charges to achieve the attenuation [60]. As observed, four SiC/C composites all display obvious frequency dispersion behaviors, which are typical signals of dipole orientation polarization due to the hysteretic reorientation of dipoles along with an applied electric field. Both the residual functional groups and defect sites in these composites can act as the polarization centers (i.e., dipoles). It is believed that interfacial polarization also contributes to dielectric loss in these composites, because there are abundant heterogeneous interfaces between carbon shells and SiC nanoparticles, and especially for SiC/C-2, SiC/C-3, and SiC/C-4, their *ε*_r_" values do not continuously decrease in high-frequency range, solidifying the formation of interfacial polarization [7]. The Debye relaxation model is an important method to study polarization loss mechanisms and can be represented by Eq. (4):

4 $$\left( {\varepsilon^{\prime} - \frac{{\varepsilon_{s} - \varepsilon_{\infty } }}{2}} \right)^{2} + \left( {\varepsilon^{\prime\prime}} \right)^{2} = \left( {\frac{{\varepsilon_{s} - \varepsilon_{\infty } }}{2}} \right)^{2}$$

where *ε*_s_ is the static dielectric constant, and *ε*_∞_ is dielectric constant at infinite frequency. According to this equation, there will be a semicircle in the Cole–Cole curve derived from *ε*_r_′ *versus ε*_r_′′ when a Debye polarization relaxation occurs, and each semicircle responds to one relaxation process under alternating electromagnetic fields. As can be seen that several semicircles will be identified in all SiC/C composites (Fig. S8), and thus there are indeed multiple polarization relaxation processes responsible for the consumption of EM energy, confirming the contribution from polarization loss. In addition, a quasi-linear tail can also be detected in SiC/C-2, SiC/C-3, and SiC/C-4, which further validates the contribution from conductivity loss [37, 61]. To illustrate the mechanism more clearly, we further quantify the specific contribution from conductivity loss (*ε*_c_′′) and polarization loss (*ε*_p_′′) in different composites based on Debye relaxation model fitted by least square method (Fig. 5d, e) [62]. It is clear that the contribution of conductivity loss will gradually decrease with increasing the frequency, and meanwhile, the strength of conductivity loss is highly consistent with the order of the conductivities of various SiC/C composites (Fig. 5f). Contrary to the change in conductivity loss, polarization loss of these composites gradually increases from 2.0 to 18.0 GHz. Noticeably, SiC/C-2 has much larger polarization loss than SiC/C-1, which can be attributed to the following two aspects: (1) the increase of SiC content brings more interfaces between carbon shells and SiC nanoparticles, thus boosting interfacial polarization; (2) the integrity of hollow structure gains more interfaces between carbon shells and wax, also intensifying interfacial polarization. However, from SiC/C-2 to SiC/C-4, polarization loss presents an overall downward trend. Possibly, the increase of SiC content inevitably compresses relative carbon content, and thus the contribution from dipole orientation polarization will be suppressed due to less residual functional groups and defect sites. This phenomenon indicates that dipole orientation polarization may play a relatively dominant role in the polarization loss of SiC/C composites with intact hollow structure, while SiC/C-4 with the highest SiC content still achieves a slight turnover in high-frequency range, implying that the contribution of interfacial polarization cannot be ignored, either.

Attenuation constant (*α*) essentially describes the amplitude attenuation of EM wave in transmission medium, while it is usually employed to feature the overall loss ability of MAMs in recent studies [63]. In terms of Eq. (5):

5 $${\upalpha } = \frac{\sqrt 2 \pi f}{c}\sqrt {\left( {\mu_{{\text{r}}}^{^{\prime\prime}} \varepsilon_{{\text{r}}}^{^{\prime\prime}} - \mu_{{\text{r}}}{\prime} \varepsilon_{{\text{r}}}{\prime} } \right) + \sqrt {\left( {\mu_{{\text{r}}}^{^{\prime\prime}} \varepsilon_{{\text{r}}}^{^{\prime\prime}} - \mu_{{\text{r}}}{\prime} \varepsilon_{{\text{r}}}{\prime} } \right)^{2} + \left( {\mu_{{\text{r}}}{\prime} \varepsilon_{{\text{r}}}^{^{\prime\prime}} + \mu_{{\text{r}}}^{^{\prime\prime}} \varepsilon_{{\text{r}}}{\prime} } \right)^{2} } }$$

frequency-dependent *α* values of different SiC/C composites are also calculated in Fig. 5g. All composites show incremental increases in *α* values from 2.0 to 18.0 GHz. It is very interesting that at a specific frequency point, *α* values display same order as that of dielectric tangent, including the intersection point between SiC/C-3 and SiC/C-4, confirming that dielectric loss is the dominant pathway for EM attenuation. Although SiC/C-2 has the largest complex permittivity, dielectric tangent, and *α* values among these composites (Fig. 5a–c, g), it still fails to produce the best microwave absorption performance (Fig. 4a, d). This is because microwave absorption performance is not just determined by intrinsic loss ability, but also correlates with impedance matching [32]. If the characteristic impedance of microwave absorption medium is mismatched with that of free space, most of EM wave will be reflected at the interface rather than being allowed to enter this medium, and thus no matter how powerful loss ability of this medium, it will not produce good microwave absorption performance. Herein, a delta value (|*Δ*|), which can be calculated using the following Eqs. (6–8), is used to estimate the matching degree of the characteristic impedance of different SiC/C/wax mixture [3, 64].

6 $$\left| \Delta \right| = \left| {{\text{sinh}}^{2} \left( {Kfd} \right) - M} \right|$$

7 $$K = \frac{{4\pi \sqrt {\mu_{{\text{r}}} \varepsilon_{{\text{r}}} } {\text{sin}}\frac{{\delta_{e} + \delta_{m} }}{2} }}{{c \cdot \cos \delta_{e} {\text{cos}}\delta_{m} }}$$

8 $$M = \frac{{4\mu_{{\text{r}}}{\prime} {\text{cos}}\delta_{e} \varepsilon_{{\text{r}}}{\prime} {\text{cos}}\delta_{m} }}{{\left( {\mu_{{\text{r}}}{\prime} {\text{cos}}\delta_{e} - \varepsilon_{{\text{r}}}{\prime} {\text{cos}}\delta_{m} } \right)^{2} + \left[ {\tan \left( {\frac{{\delta_{m} }}{2} - \frac{{\delta_{e} }}{2}} \right)} \right]^{2} \left( {\mu_{{\text{r}}}{\prime} {\text{cos}}\delta_{e} + \varepsilon_{{\text{r}}}{\prime} {\text{cos}}\delta_{m} } \right)^{2} }}$$

From Figs. 5h and S9, one can find that the coverage ratio with desirable |*∆*| values of the mixture with SiC/C-1, SiC/C-2, SiC/C-3, and SiC/C-4 are 25.9%, 42.0%, 58.2%, and 34.8%, respectively. It is undoubted that the mixture with SiC/C-3 as the filler harvests the best impedance matching, and these results also explain why SiC/C-3 does not generate the strongest loss ability but produce the best microwave absorption performance.

In addition to the effect of compositional changes on the microwave absorption properties, the mass fraction of SiC/C in wax is also another crucial parameter. Therefore, SiC/C-3 with the best microwave absorption performance is taken as a representative sample to explore the relationship between mass fraction and microwave absorption performance. 2D RL mapping and RL curves below − 10 dB of SiC/C-3 with different mass fraction (30% and 40%) are plotted in Fig. S10. As can be seen, the *RL*_min_ values are − 17.3 and − 26.3 dB, respectively, for mass fractions of 30% and 40%, which are obviously weaker than that generated with the mass fraction of 35% (Fig. 4b, e). More importantly, the maximum EABs generated with the mass fraction of 30% and 40% (4.2 and 3.8 GHz) are also much narrower than 5.1 GHz (35%). EM parameters of SiC/C-3 with different mass fraction are further analyzed to investigate the reason for different microwave absorption performance with the change of mass fraction. As depicted in Fig. S11, both *ε*_r_′ and *ε*_r_" values of SiC/C-3 in whole frequency range are monotonously increased with the mass fraction, representing the gradual improvement in storage and dissipation abilities of electric energy, while *μ*_r_′ and *μ*_r_" values seem insensitive to the mass fraction, again verifying the attenuation of EM wave is overwhelmingly dependent on dielectric loss. From these results, one can speculate that the inferior microwave absorption with the mass fraction of 30% is mainly attributed to insufficient dielectric loss ability generated by small *ε*_r_′ and *ε*_r_" values. However, a relatively high mass fraction (i.e. 40%) does not bring better microwave absorption performance, which is caused by the deterioration in impedance matching. This situation is very similar to that of SiC/C-4. Therefore, the mass fraction of 35% is a relatively suitable filler loading for hollow SiC/C composites in current study.

Based on the analysis above, we attempt to illustrate the reasons for good microwave absorption performance of SiC/C-3 in Fig. 5i. First, the establishment of hollow structure remarkably increase the specific volume of SiC/C-3, which makes it easy to construct conductive networks in wax matrix, generating considerable contribution from conductivity loss. Second, although the mesoporous structure cannot directly induce multiple reflection of incident EM wave due to their small size, the cavity of these microspheres reaches the micron scale, and they may gain an opportunity to achieve multiple reflection. In addition, the interspaces among SiC/C microspheres can also intensify the multiple reflection of EM wave, thus promoting the consumption of EM energy. Third, the embedment of SiC nanoparticles in carbon shells creates abundant heterogeneous interfaces and results in the uneven accumulation of free charges at those interfaces, and they will generate a reverse internal electric field in response to an external EM field, forming a capacitor-like configuration and thus increasing their interfacial polarization loss. Fourth, both carbon shells and SiC nanoparticles in SiC/C-3 can provide numerous sites (residual functional groups, defect sites, intrinsic dipoles) to act as the active sites of dipole orientation polarization along with an alternating EM field, but in view of the decrease in polarization loss from SiC/C-2 to SiC/C-4, residual functional groups and defect sites in carbon shells may be the main force to afford dipole orientation polarization. It has to be pointed out that these four advantages are actually also applicable to SiC/C-2 and SiC/C-4, but compared with them, SiC/C-3 has more proper composition, and thus it can bring better impedance matching and further produce the best microwave absorption performance. In other words, such good microwave absorption performance of SiC/C-3 benefits from the synergy of compositional and hollow engineering.

### Effect of Pyrolysis Temperature on Microwave Absorption Performance

Apart from adjusting the molar ratio of TEOS/resorcinol, the pyrolysis temperature may also produce significant impacts on EM properties of SiC/C composites, and thus two additional samples are further prepared at 700 and 900 °C in terms of the same molar ratio of TEOS/resorcinol in SiC/C-3, which are denoted as SiC/C-3-700 and SiC/C-3-900, respectively. As shown in Fig. S12a, SiC/C-3-700 and SiC/C-3-900 give almost identical characteristic diffraction peaks to those of SiC/C-3 from 800 °C, while the intensity of the peak at 35.4° in SiC/C-3-900 becomes relatively stronger. These results suggest that SiC nanoparticles are also generated in these two samples, and although high pyrolysis temperature seems helpful to improve the crystallinity of SiC nanoparticles, it still cannot achieve the complete graphitization of carbon matrix. The specific carbon contents in SiC/C-3-700 and SiC/C-3-900 are deduced as 66.5% and 63.0%, respectively, based on air-atmosphere TG curves (Fig. S12b). Of note is that the onset of the temperature for drastic weight decrease (i.e., carbon combustion) slightly shifts to high temperature, which implies that the relative graphitization degree of carbon components in these composites may be gradually enhanced with increasing the pyrolysis temperature. Raman spectra reveal that *I*_D_/*I*_G_ values for SiC/C-3-700 and SiC/C-3-900 are 0.91 and 1.10, respectively (Fig. S12c). This phenomenon further validates the improvement of relative graphitization degree of carbon components from SiC/C-3700 to SiC/C-3900, because Ferrari and Robertson ever represented that such a change trend could be attributed to the formation of tiny nanocrystalline domains in amorphous carbon matrix [32]. In addition, N_2_ adsorption–desorption isotherms and SEM images indicate that the change of pyrolysis temperature does not affect the microstructure and morphology of SiC/C composites obviously (Figs. S12d and S13), and the slight decrease in the pore volume of SiC/C-3900 may be attributed to the fact that high pyrolysis temperature breaks a very small number of SiC/C microspheres, as indicated by some sporadic fragments in SEM image (Fig. S13b). These results disclose that pyrolysis temperature has a greater impact on the content and relative graphitization degree of carbon components in the composites than that on microstructure, and thus the EM properties of SiC/C composites will also be changed. As shown in Fig. S14, SiC/C-3700 present the lowest *ε*_r_′ and *ε*_r_″ values among these samples, whose *ε*_r_′ and *ε*_r_″ values gradually change from 9.2 and 2.7 at 2.0 GHz to 7.1 and 2.7 at 18.0 GHz, respectively. By comparison, *ε*_r_′ values of SiC/C-3–900 are significantly increased to 14.4 at 2.0 GHz and 10.3 at 18.0 GHz, respectively, and the corresponding *ε*_r_″ values are also raised to 7.6 at 2.0 GHz and 4.9 at 18.0 GHz, respectively. It is obvious that high pyrolysis temperature indeed favors large *ε*_r_' and *ε*_r_" values at a given frequency point, indicating that their dielectric loss ability is also gradually enhanced from SiC/C-3700 to SiC/C-3900. The measured conductivities of the mixture with SiC/C-3–700 and SiC/C-3-900 are 4.1 × 10^–4^ and 7.7 × 10^–4^ S cm^−1^, respectively (Fig. S15), indicating that the improved graphitization degree enhances electronic transmission capability. That is to say, there will be stronger leakage current under applied EM field, which also consolidate conductivity loss and total dielectric loss of SiC/C composites. As expected, SiC/C-3–700 and SiC/C-3–900 indeed give different microwave absorption performance from that of SiC/C-3 (Fig. S16), whose *RL*_min_ intensities are − 17.6 dB (10.4 GHz, 2.7 mm) and − 23.7 dB (12.9 GHz, 1.8 mm), respectively, and the corresponding EABs are 4.2 GHz (13.8–18 GHz, 1.8 mm) and 4.3 GHz (13.7–18 GHz, 1.5 mm), respectively. Both *RL*_min_ intensities and EABs, as well as the coverages of qualified absorption of SiC/C-3-700 and SiC/C-3–900 are inferior to those of SiC/C-3. After analyzing their *α* and |*Δ*| values (Fig. S17), one can safely conclude that the weak attenuation ability of SiC/C-3-700 and the degraded impedance matching of SiC/C-3-900 are the key points for their insufficient microwave absorption. That is to say, 800 °C may be an optimum pyrolysis temperature for the preparation of SiC/C composites.

### Environmental Tolerance and Radar Stealth Performance of SiC/C Composites

**Fig. 6:**
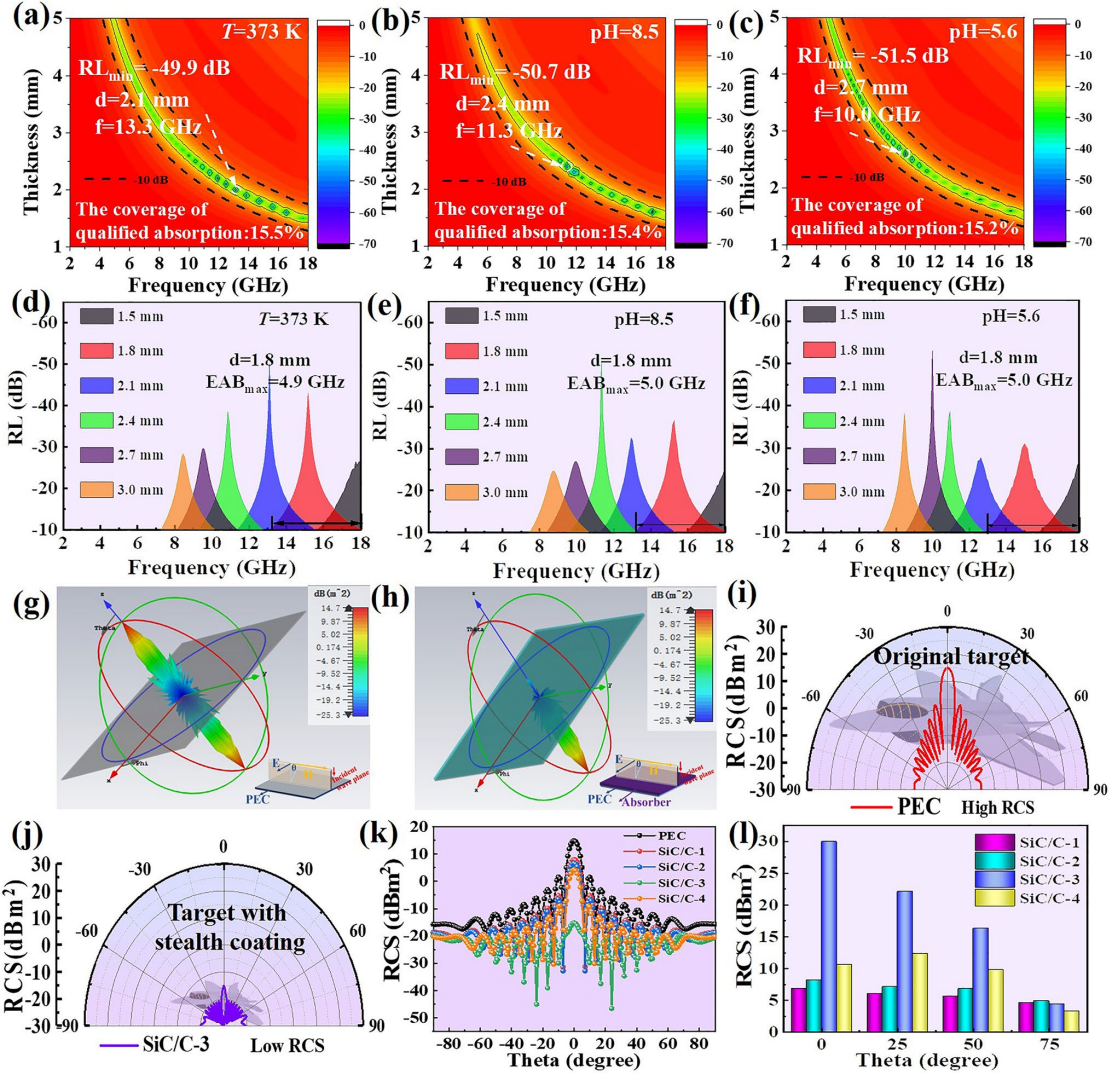
2D RL maps for **a** *T* = 373 K, **b** pH = 8.5, and **c** pH = 5.6. RL curves below -10 dB at given absorber thickness for **d** *T* = 373 K, **e** pH = 8.5, and **f** pH = 5.6. **g** 3D radar wave scattering signals of PEC and **h** PEC coated with SiC/C-3. **i** RCS simulated curves and **k** RCS in polar coordinate system of PEC and **l** PEC plate coated with SiC/C-3 at 14.0 GHz. **j** RCS reduction values of PEC and PEC coated with different SiC/C composites

As mentioned above, environmental tolerance is an important indicator to evaluate the practical prospect of MAMs [14]. Therefore, we treat SiC/C-3 under three different conditions (*T* = 373 K, pH = 8.5, and pH = 5.6 solution) for 120 h to simulate its application in natural environments, which corresponds to sun exposure, acid rain, and seawater, respectively. As observed, as compared to the untreated SiC/C-3 (Fig. S18), these treatments indeed induce slight changes in relative complex permittivity, which suggest that natural environments may produce more or less impacts on its microwave absorption characteristics. After plotting the frequency-dependent RL maps of SiC/C-3 treated under different conditions, one can find that there is a moderate decrease in *RL*_min_ intensity from − 60.8 to − 49.9 dB (*T* = 373 K), to − 50.7 dB (pH = 8.5), and to -51.5 dB (pH = 5.6), respectively (Fig. 6a–c). However, the EAB of SiC/C-3 just narrows 0.1–0.2 GHz (Fig. 6d–f), and the coverages of EAB in the frequency range of 2.0–18.0 GHz remains above 15.0% (Fig. 6a–c). Even if we treat SiC/C-3 in a strong acidic solution (0.2 mol L^−1^ of HCl solution) for 120 h, it can also maintain good microwave absorption performance (Fig. S19), and in contrast, some conventional magnetic MAMs, such as Fe, Fe_3_O_4_ and Ni, will be intensively corroded (Fig. S20). All these results clearly validate the bright prospect of practical application for SiC/C-3. In addition, the radar stealth performance of SiC/C-3 is also deduced by 3D far-field radiation photos and 2D RCS distributions with different detection angle of perfect electric conductor (PEC) plate and MAMs-coated PEC plates (Fig. 6g–l). It is worth noting that the radar scattering signal intensity of the original PEC plate is quite pronounced, while it becomes extremely weak after being coated by SiC/C-3, displaying a strong EM attenuation effect (Fig. 6g–j). As shown in Fig. 6k, l, the largest RCS reduction values of the PEC coated by SiC/C-1, SiC/C-2, SiC/C-3, and SiC/C-4 are 6.9, 8.2, 29.9 and 12.3 dB m^2^ at the pitching angels changing from -90° to 90°, respectively. This result shows that all the SiC/C samples contribute to the reduction of RCS values, but SiC/C-3 has the highest RCS reduction values among them, that is, it can achieve the better radar wave attenuation performance. The above analysis demonstrates that SiC/C-3 composites exhibit good environmental stability and radar stealth performance, displaying excellent potential for practical application.

## Conclusions

In summary, hollow SiC/C microspheres with controllable composition have been successfully synthesized through a heterogeneous interfacial anti-interaction strategy. The molar ratio of TEOS/resorcinol not only plays a crucial role in the creation of hollow structure, but also affords the effect of compositional regulation. Results indicated that the combination of compositional and structural engineering is a highly effective way to optimize EM properties and achieve strong microwave absorption performance. Especially for the composite with the content of SiC nanoparticles at 35.4%, the minimum RL intensity and the maximum effective absorption bandwidth can reach − 60.8 dB and 5.1 GHz, respectively. The mechanism investigation reveals that conductivity loss and interfacial polarization, and dipole orientation polarization, as well as hollow structure, are together responsible for powerful attenuation ability. In addition, the environmental tolerance tests and RCS simulation indicate that hollow SiC/C microspheres have bright prospects in practical applications.

## Supplementary Information

Below is the link to the electronic supplementary material.

Supplementary file1 (PDF 2062 KB)

## References

[1] Z. Cheng, R. Wang, Y. Cao, Z. Cai, Z. Zhang et al., Intelligent off/on switchable microwave absorption performance of reduced graphene oxide/VO_2_ composite aerogel. Adv. Funct. Mater. **32**, 2205160 (2022). 10.1002/adfm.202205160

[2] B. Zhao, Z. Yan, Y. Du, L. Rao, G. Chen et al., High-entropy enhanced microwave attenuation in titanate perovskites. Adv. Mater. **35**, 2210243 (2023). 10.1002/adma.202210243 36606342 10.1002/adma.202210243

[3] L. Gai, H. Zhao, F. Wang, P. Wang, Y. Liu et al., Advances in core—shell engineering of carbon-based composites for electromagnetic wave absorption. Nano Res. **15**, 9410–9439 (2022). 10.1007/s12274-022-4695-6

[4] J. Lin, J. Qiao, H. Tian, L. Li, W. Liu et al., Ultralight, hierarchical metal–organic framework derivative/graphene hybrid aerogel for electromagnetic wave absorption. Adv. Compos. Hybrid Mater. **6**, 177 (2023). 10.1007/s42114-023-00762-w

[5] B. Zhao, Z. Bai, H. Lv, Z. Yan, Y. Du et al., Self-healing liquid metal magnetic hydrogels for smart feedback sensors and high-performance electromagnetic shielding. Nano-Micro Lett. **15**, 79 (2023). 10.1007/s40820-023-01043-3 10.1007/s40820-023-01043-3 PMC10066054 37002442

[6] M. Qin, L. Zhang, H. Wu, Dielectric loss mechanism in electromagnetic wave absorbing materials. Adv. Sci. **9**, 2105553 (2022). 10.1002/advs.202105553 10.1002/advs.202105553 PMC8981909 35128836

[7] J. Liu, J. Tao, L. Gao, X. He, B. Wei et al., Morphology-size synergy strategy of SiC@C nanoparticles towards lightweight and efficient microwave absorption. Chem. Eng. J. **433**, 134484 (2022). 10.1016/j.cej.2021.134484

[8] C. Wang, Y. Liu, Z. Jia, W. Zhao, G. Wu, Multicomponent nanoparticles synergistic one-dimensional nanofibers as heterostructure absorbers for tunable and efficient microwave absorption. Nano-Micro Lett. **15**, 13 (2022). 10.1007/s40820-022-00986-3 10.1007/s40820-022-00986-3 PMC9755410 36520259

[9] Y. Du, Z. Yan, W. You, Q. Men, G. Chen et al., Balancing MXene surface termination and interlayer spacing enables superior microwave absorption. Adv. Funct. Mater. **33**, 2301449 (2023). 10.1002/adfm.202301449

[10] F. Wang, Y. Liu, H. Zhao, L. Cui, L. Gai et al., Controllable seeding of nitrogen-doped carbon nanotubes on three-dimensional Co/C foam for enhanced dielectric loss and microwave absorption characteristics. Chem. Eng. J. **450**, 138160 (2022). 10.1016/j.cej.2022.138160

[11] D. Liu, Y. Du, P. Xu, F. Wang, Y. Wang et al., Rationally designed hierarchical N-doped carbon nanotubes wrapping waxberry-like Ni@C microspheres for efficient microwave absorption. J. Mater. Chem. A **9**, 5086–5096 (2021). 10.1039/D0TA10942H

[12] Y. Xiong, L. Xu, C. Yang, Q. Sun, X. Xu, Implanting FeCo/C nanocages with tunable electromagnetic parameters in anisotropic wood carbon aerogels for efficient microwave absorption. J. Mater. Chem. A **8**, 18863–18871 (2020). 10.1039/D0TA05540A

[13] L. Liu, N. He, T. Wu, P. Hu, G. Tong, Co/C/Fe/C hierarchical flowers with strawberry-like surface as surface plasmon for enhanced permittivity, permeability, and microwave absorption properties. Chem. Eng. J. **355**, 103–108 (2019). 10.1016/j.cej.2018.08.131

[14] Z. Lou, Q. Wang, U.I. Kara, R.S. Mamtani, X. Zhou et al., Biomass-derived carbon heterostructures enable environmentally adaptive wideband electromagnetic wave absorbers. Nano-Micro Lett. **14**, 11 (2021). 10.1007/s40820-021-00750-z 10.1007/s40820-021-00750-z PMC8643388 34862949

[15] C. Zheng, M. Ning, Z. Zou, G. Lv, Q. Wu et al., Two birds with one stone: broadband electromagnetic wave absorption and anticorrosion performance in 2–18 GHz for Prussian blue analog derivatives aimed for practical applications. Small **19**, 2208211 (2023). 10.1002/smll.202208211 37078912 10.1002/smll.202208211

[16] S. Chen, W. Li, X. Li, W. Yang, One-dimensional SiC nanostructures: designed growth, properties, and applications. Prog. Mater. Sci. **104**, 138–214 (2019). 10.1016/j.pmatsci.2019.04.004

[17] B. Hu, L. Gai, Y. Liu, P. Wang, S. Yu et al., State-of-the-art in carbides/carbon composites for electromagnetic wave absorption. iScience **26**, 107876 (2023). 10.1016/j.isci.2023.107876 37767003 10.1016/j.isci.2023.107876 PMC10520892

[18] Z. Cai, L. Su, H. Wang, M. Niu, H. Gao et al., Hydrophobic SiC@C nanowire foam with broad-band and mechanically controlled electromagnetic wave absorption. ACS Appl. Mater. Interfaces **12**, 8555–8562 (2020). 10.1021/acsami.9b20636 31985205 10.1021/acsami.9b20636

[19] Y. Du, Advances in carbon-based microwave absorbing materials. Materials **15**, 1359 (2022). 10.3390/ma15041359 35207899 10.3390/ma15041359 PMC8877884

[20] Y. Cheng, L. Hu, K. Zhang, J. Fan, Facile synthesis of hollow SiC/C nanospheres for high-performance electromagnetic wave absorption. Carbon **215**, 118391 (2023). 10.1016/j.carbon.2023.118391

[21] B. Huang, J. Yue, B. Fan, Y. Liu, X. Huang, Vertical carbon nanotubes arrays with controlled morphology on silicon carbide fibers for electromagnetic wave absorption. Ceram. Int. **48**, 19375–19381 (2022). 10.1016/j.ceramint.2022.03.232

[22] Z. Wu, H.-W. Cheng, C. Jin, B. Yang, C. Xu et al., Dimensional design and core-shell engineering of nanomaterials for electromagnetic wave absorption. Adv. Mater. **34**, 2107538 (2022). 10.1002/adma.202107538 34755916 10.1002/adma.202107538

[23] C. Zhang, Z. Wu, C. Xu, B. Yang, L. Wang et al., Hierarchical Ti_3_C_2_T_*x*_ MXene/carbon nanotubes hollow microsphere with confined magnetic nanospheres for broadband microwave absorption. Small **18**, 2104380 (2022). 10.1002/smll.202104380 10.1002/smll.202104380 34914181

[24] Y. Hou, H. Yuan, X. Qu, H. Chen, L. Li, Synthesis and high-performance electromagnetic wave absorption of SiC@C composites. Mater. Lett. **209**, 90–93 (2017). 10.1016/j.matlet.2017.07.114

[25] H. Xu, G. Zhang, Y. Wang, M. Ning, B. Ouyang et al., Size-dependent oxidation-induced phase engineering for MOFs derivatives via spatial confinement strategy toward enhanced microwave absorption. Nano-Micro Lett. **14**, 102 (2022). 10.1007/s40820-022-00841-5 10.1007/s40820-022-00841-5 PMC9005575 35412156

[26] H. Zhao, F. Wang, L. Cui, X. Xu, X. Han et al., Composition optimization and microstructure design in MOFs-derived magnetic carbon-based microwave absorbers: a review. Nano-Micro Lett. **13**, 208 (2021). 10.1007/s40820-021-00734-z 10.1007/s40820-021-00734-z PMC8505592 34633562

[27] H. Zhao, X. Xu, Y. Wang, D. Fan, D. Liu et al., Heterogeneous interface induced the formation of hierarchically hollow carbon microcubes against electromagnetic pollution. Small **16**, 2003407 (2020). 10.1002/smll.202003407 10.1002/smll.202003407 33015974

[28] X.-F. Zhang, Z. Chen, Y. Feng, J. Qiu, J. Yao, Low-temperature transformation of C/SiO_2_ nanocomposites to β-SiC with high surface area. ACS Sustainable Chem. Eng. **6**, 1068–1073 (2018). 10.1021/acssuschemeng.7b03375

[29] Y. Du, J. Wang, C. Cui, X. Liu, X. Wang et al., Pure carbon microwave absorbers from anion-exchange resin pyrolysis. Synth. Met. **160**, 2191–2196 (2010). 10.1016/j.synthmet.2010.08.008

[30] J.-P. Chen, Y.-F. Du, Z.-F. Wang, L.-L. Liang, H. Jia et al., Anchoring of SiC whiskers on the hollow carbon microspheres inducing interfacial polarization to promote electromagnetic wave attenuation capability. Carbon **175**, 11–19 (2021). 10.1016/j.carbon.2020.12.073

[31] M. Zhang, H. Lin, S. Ding, T. Wang, Z. Li et al., Net-like SiC@C coaxial nanocable towards superior lightweight and broadband microwave absorber. Compos. Part B Eng. **179**, 107525 (2019). 10.1016/j.compositesb.2019.107525

[32] F. Wang, Y. Liu, R. Feng, X. Wang, X. Han et al., A “win–win” strategy to modify Co/C foam with carbon microspheres for enhanced dielectric loss and microwave absorption characteristics. Small **19**, 2303597 (2023). 10.1002/smll.202303597 10.1002/smll.202303597 37528502

[33] N. Wang, W. Ma, Z. Ren, Y. Du, P. Xu et al., Prussian blue analogues derived porous nitrogen-doped carbon microspheres as high-performance metal-free peroxymonosulfate activators for non-radical-dominated degradation of organic pollutants. J. Mater. Chem. A **6**, 884–895 (2018). 10.1039/C7TA08472B

[34] Y. Du, T. Liu, B. Yu, H. Gao, P. Xu et al., The electromagnetic properties and microwave absorption of mesoporous carbon. Mater. Chem. Phys. **135**, 884–891 (2012). 10.1016/j.matchemphys.2012.05.074

[35] S. Hou, Y. Wang, F. Gao, H. Yang, F. Jin et al., A novel approach to electromagnetic wave absorbing material design: Utilizing nano-antenna arrays for efficient electromagnetic wave capture. Chem. Eng. J. **471**, 144779 (2023). 10.1016/j.cej.2023.144779

[36] H. Wang, Q. An, Z. Xiao, Y. Tong, L. Guo et al., Marine polysaccharide-based electromagnetic absorbing/shielding materials: design principles, structure, and properties. J. Mater. Chem. A **10**, 17023–17052 (2022). 10.1039/D2TA03529D

[37] L. Gai, Y. Zhao, G. Song, Q. An, Z. Xiao et al., Construction of core-shell PPy@MoS_2_ with nanotube-like heterostructures for electromagnetic wave absorption: assembly and enhanced mechanism. Compos. Part A Appl. Sci. Manuf. **136**, 105965 (2020). 10.1016/j.compositesa.2020.105965

[38] M. Han, X. Yin, Z. Hou, C. Song, X. Li et al., Flexible and thermostable graphene/SiC nanowire foam composites with tunable electromagnetic wave absorption properties. ACS Appl. Mater. Interfaces **9**, 11803–11810 (2017). 10.1021/acsami.7b00951 28317374 10.1021/acsami.7b00951

[39] Y. Jiang, Y. Chen, Y. J. Liu, G. X. Sui, Lightweight spongy bone-like graphene@SiC aerogel composites for high-performance microwave absorption. Chem. Eng. J. **337**, 522–531 (2018). 10.1016/j.cej.2017.12.131

[40] J. Qian, B. Du, M. Cai, C. He, X. Wang et al., Preparation of SiC nanowire/carbon fiber composites with enhanced electromagnetic wave absorption performance. Adv. Eng. Mater. **23**, 2100434 (2021). 10.1002/adem.202100434

[41] S. Xiao, H. Mei, D. Han, K.G. Dassios, L. Cheng, Ultralight lamellar amorphous carbon foam nanostructured by SiC nanowires for tunable electromagnetic wave absorption. Carbon **122**, 718–725 (2017). 10.1016/j.carbon.2017.07.023

[42] S. Xie, G. Q. Jin, S. Meng, Y. W. Wang, Y. Qin et al., Microwave absorption properties of *in situ* grown CNTs/SiC composites. J. Alloys Compd. **520**, 295–300 (2012). 10.1016/j.jallcom.2012.01.050

[43] X. Ye, Z. Chen, S. Ai, B. Hou, J. Zhang et al., Novel three-dimensional SiC/melamine-derived carbon foam-reinforced SiO_2_ aerogel composite with low dielectric loss and high impedance matching ratio. ACS Sustain. Chem. Eng. **7**, 2774–2783 (2019). 10.1021/acssuschemeng.8b05966

[44] K. Zhao, F. Ye, L. Cheng, R. Liu, J. Liang et al., Synthesis of embedded ZrC-SiC-C microspheres via carbothermal reduction for thermal stability and electromagnetic wave absorption. Appl. Surf. Sci. **591**, 153105 (2022). 10.1016/j.apsusc.2022.153105

[45] B. Mao, X. Xia, R. Qin, D. Xu, X. Wang et al., Synthesis and microwave absorption properties of multilayer SiC/C foam with alternating distribution of C and SiC. J. Alloys Compd. **879**, 160440 (2021). 10.1016/j.jallcom.2021.160440

[46] Z. Hou, J. Xue, H. Wei, X. Fan, F. Ye et al., Tailorable microwave absorption properties of RGO/SiC/CNT nanocomposites with 3D hierarchical structure. Ceram. Int. **46**, 18160–18167 (2020). 10.1016/j.ceramint.2020.04.137

[47] S. Singh, A. Kumar, D. Singh, Enhanced microwave absorption performance of SWCNT/SiC composites. J. Electron. Mater. **49**, 7279–7291 (2020). 10.1007/s11664-020-08460-9

[48] R. Wu, Z. Yang, M. Fu, K. Zhou, *In-situ* growth of SiC nanowire arrays on carbon fibers and their microwave absorption properties. J. Alloys Compd. **687**, 833–838 (2016). 10.1016/j.jallcom.2016.06.106

[49] X. Ye, Z. Chen, S. Ai, B. Hou, J. Zhang et al., Porous SiC/melamine-derived carbon foam frameworks with excellent electromagnetic wave absorbing capacity. J. Adv. Ceram. **8**, 479–488 (2019). 10.1007/s40145-019-0328-2

[50] X. Ye, Z. Chen, S. Ai, B. Hou, J. Zhang et al., Synthesis and microwave absorption properties of novel reticulation SiC/Porous melamine-derived carbon foam. J. Alloys Compd. **791**, 883–891 (2019). 10.1016/j.jallcom.2019.03.384

[51] Y. Zhang, J. Chen, D. Yan, S. Wang, G. Li et al., Conversion of silicon carbide fibers to continuous graphene fibers by vacuum annealing. Carbon **182**, 435–444 (2021). 10.1016/j.carbon.2021.06.043

[52] Y. Zhang, Y. Zhao, Q. Chen, Y. Hou, Q. Zhang et al., Flexible SiC-CNTs hybrid fiber mats for tunable and broadband microwave absorption. Ceram. Int. **47**, 8123–8132 (2021). 10.1016/j.ceramint.2020.11.167

[53] P. Wang, L. Gai, B. Hu, Y. Liu, F. Wang et al., Topological MOFs deformation for the direct preparation of electromagnetic functionalized Ni/C aerogels with good hydrophobicity and thermal insulation. Carbon **212**, 118132 (2023). 10.1016/j.carbon.2023.118132

[54] L. Cui, Y. Wang, X. Han, P. Xu, F. Wang et al., Phenolic resin reinforcement: a new strategy for hollow NiCo@C microboxes against electromagnetic pollution. Carbon **174**, 673–682 (2021). 10.1016/j.carbon.2020.10.070

[55] D. Liu, R. Qiang, Y. Du, Y. Wang, C. Tian et al., Prussian blue analogues derived magnetic FeCo alloy/carbon composites with tunable chemical composition and enhanced microwave absorption. J. Colloid Interface Sci. **514**, 10–20 (2018). 10.1016/j.jcis.2017.12.013 29227802 10.1016/j.jcis.2017.12.013

[56] Z.N. Wing, B. Wang, J.W. Halloran, Permittivity of porous titanate dielectrics. J. Am. Ceram. Soc. **89**, 3696–3700 (2006). 10.1111/j.1551-2916.2006.01323.x

[57] D. Liu, Y. Du, F. Wang, Y. Wang, L. Cui et al., MOFs-derived multi-chamber carbon microspheres with enhanced microwave absorption. Carbon **157**, 478–485 (2020). 10.1016/j.carbon.2019.10.056

[58] T. Zhao, Z. Jia, Y. Zhang, G. Wu, Multiphase molybdenum carbide doped carbon hollow sphere engineering: the superiority of unique double-shell structure in microwave absorption. Small **19**, e2206323 (2023). 10.1002/smll.202206323 36436944 10.1002/smll.202206323

[59] R. Qiang, Y. Du, Y. Wang, N. Wang, C. Tian et al., Rational design of yolk-shell C@C microspheres for the effective enhancement in microwave absorption. Carbon **98**, 599–606 (2016). 10.1016/j.carbon.2015.11.054

[60] Z. Gao, Z. Ma, D. Lan, Z. Zhao, L. Zhang et al., Synergistic polarization loss of MoS_2_-based multiphase solid solution for electromagnetic wave absorption. Adv. Funct. Mater. **32**, 2112294 (2022). 10.1002/adfm.202112294

[61] K.S. Cole, R.H. Cole, Dispersion and absorption in dielectrics I: alternating current characteristics. J. Chem. Phys. **9**, 341–351 (1941). 10.1063/1.1750906

[62] B. Zhao, Y. Du, H. Lv, Z. Yan, H. Jian et al., Liquid-metal-assisted programmed galvanic engineering of core–shell nanohybrids for microwave absorption. Adv. Funct. Mater. **33**, 2302172 (2023). 10.1002/adfm.202302172

[63] Y. Liu, C. Tian, F. Wang, B. Hu, P. Xu et al., Dual-pathway optimization on microwave absorption characteristics of core–shell Fe_3_O_4_@C microcapsules: composition regulation on magnetic core and MoS_2_ nanosheets growth on carbon shell. Chem. Eng. J. **461**, 141867 (2023). 10.1016/j.cej.2023.141867

[64] D. Liu, Y. Du, P. Xu, N. Liu, Y. Wang et al., Waxberry-like hierarchical Ni@C microspheres with high-performance microwave absorption. J. Mater. Chem. C **7**, 5037–5046 (2019). 10.1039/C9TC00771G

