# Supplementary material for: Compositional and Hollow Engineering of Silicon Carbide/Carbon Microspheres as High-Performance Microwave Absorbing Materials with Good Environmental Tolerance
Source: Nanomicro Lett. 2024 Apr 2;16:167. doi: 10.1007/s40820-024-01369-6 (PMC10987424; doi:10.1007/s40820-024-01369-6)
Supplement: Supplementary file 1 — Supplementary file1 (PDF 2062 KB) [file 40820_2024_1369_MOESM1_ESM.pdf]

Supporting Information for

Microspheres as High-Performance Microwave Absorbing Materials with Good Environmental Tolerance

Lixue Gai[1], Yahui Wang[2,*], Pan Wan[1], Shuping Yu[1], Yongzheng Chen[1], Xijiang Han[1], Ping Xu[1] and Yunchen Du[1,*]

[1]MIIT Key Laboratory of Critical Materials Technology for New Energy Conversion and Storage, School of Chemistry and Chemical Engineering, Harbin Institute of Technology, Harbin 150001, P. R. China

[2]Anhui Provincial Laboratory of Advanced Laser Technology, College of Electronic Engineering, National University of Defense Technology, Hefei 230037, P. R. China

*Corresponding authors. E-mail: yunchendu@hit.edu.cn (Yunchen Du); wangyahui22@nudt.edu.cn (Yahui Wang)

S1 Materials Characterization

The morphology and structure of all composites were investigated using a scanning electron microscope (SEM, HELIOS NanoLab 600i) with an energy-dispersive X-ray (EDX) detector and a transmission electron microscope (TEM, JEOL JEM-3000F). X-ray diffraction (XRD, ESCALAB210) with a scanning range of 10°-90° and X-ray photoelectron spectroscopy (XPS, Kratos Axis Supra) were carried out to analyze the crystallographic structures and phase composition. The Brunauer-Emmett-Teller (BET) surface area was calculated from nitrogen adsorption isotherms collected at 77 K using a Tristar-3020 instrument. Thermogravimetric analysis (TGA) was performed by a TGA Q500 under a N_2 atmosphere from room temperature to 800 °C at a rate of 10 °C/min. Raman spectrum was determined by a Raman microscopy (Renishaw PLC) using laser excitation at 532 nm. Fouriertransform infrared (FTIR) spectra were recorded on a Nicolet AVATAR FT-IR360 spectrophotometer. The conductivity of the composite was measured using a four-probe resistivity meter (RTS-9, Guangzhou 4-probes Technology Co., Ltd., China).

S2 Electromagnetic Parameter Measurement

The electromagnetic measurements, including the complex relative permittivity ($\varepsilon_r = \varepsilon_r'$-j$\varepsilon_r''$) and permeability ($\mu_r$=$\mu_r'$-j$\mu_r''$), were performed by the coaxial-line method on a vector network analyzer (Agilent N5230A) in the frequency range of 2.0-18.0 GHz. The standard coaxial ring (φ_{in} = 3.04 mm, φ_{out} = 7.00 mm) was prepared by mixing solid wax with as-prepared SiC/C composites in a mass fraction of 35%. The electromagnetic absorption efficiency in the studied frequency range was evaluated using the RL (dB) associated with the above measured electromagnetism parameter,

and the minimum RL usually corresponds to the largest absorption efficiency. The RL values for different matching thicknesses were calculated according to the following formulas:

$$RL=20\log|(Z_{in}-Z_o)/(Z_{in}+Z_o)| \qquad (S1)$$

$$Z_{in}=Z_o(\mu_r/\varepsilon_r)^{1/2}\tanh[j(2\pi fd)/c(\mu_r\varepsilon_r)^{1/2}] \qquad (S2)$$

In which, Z_{in} and Z_o represent the input and free-space impedances, respectively. f, d and c were the frequency, thickness of the sample, and speed of light in vacuum.

NOTE: both EM parameters and microwave absorption properties are from the mixture of SiC/C composites and wax matrix, but we briefly describe them with SiC/C composites in this study because wax herein is organic binder and wave-transparent.

S3 CST Simulation

The Computer Simulation Technology (CST) Microwave Studio were performed to evaluate and predict the scattering problem of absorbing materials in specific frequency electromagnetic waves by integral equation solver. In general, the simulation model was composed of two square shape layers with the overall size of 180 × 180 mm^2. The bottom layer, with a thickness of 1.5 mm, was a backing plate of perfect electric conductor (PEC), which was chosen due to its lossless properties to the incident electromagnetic waves. The upper layer was an absorber layer with the thickness of 2.0 mm, and their electromagnetic parameters are obtained by fitting with the dispersion model for the input measured electromagnetic parameters. In the simulation process, the linear polarized plane electromagnetic waves direction was set for the positive direction of the Z axis to the negative direction of the Z axis. Meanwhile, the electric field and the magnetic field polarization propagation direction along X axis and Y axis, respectively. The far-field open boundaries in all directions were to simulate the free space environment, and the field monitoring frequency was 14.0 GHz. The RCS values was obtained by using the following equation:

$$\sigma(\mathrm{dB\ m^2}) = 10\log\left(\frac{4\pi S}{\lambda^2}\left|\frac{E_s}{E_i}\right|^2\right) \qquad (S3)$$

where S and λ represent the area of the target object simulation model and the wavelength of the electromagnetic wave, respectively, E_s and E_i refer to the electric field intensity of the transmitted waves and the electric field intensity of the received waves, respectively.

Supplementary Figures and Tables

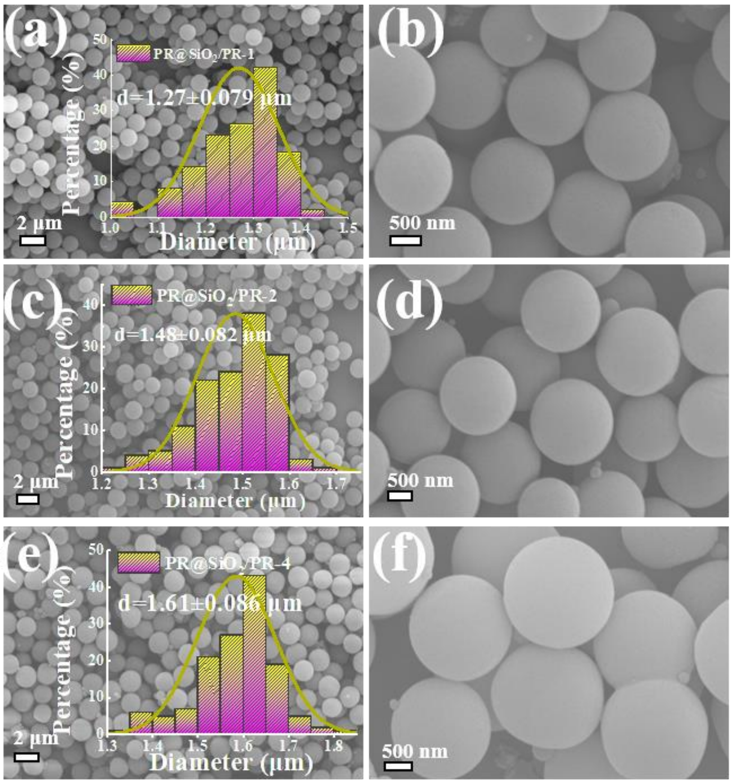

Fig. S1 a-b SEM images of PR@SiO_2/PR-1, **c-d** PR@SiO_2/PR-2 and **e-f** PR@SiO_2/PR-4 (The insets are the corresponding statistical distribution of diameters)

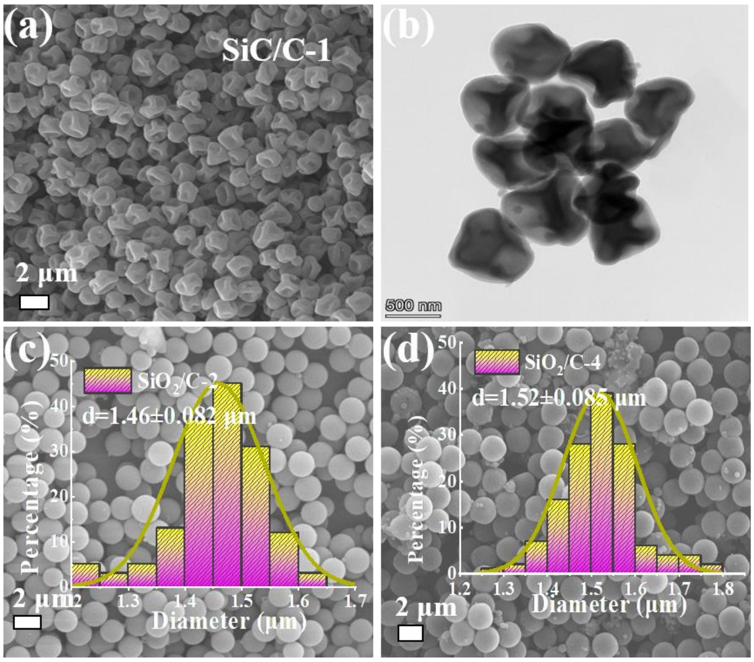

Fig. S2 a SEM images and **b** TEM images of SiO_2/C-1. **c** SEM images of SiO_2/C-2 and **d** SiO_2/C-4 (The insets are the corresponding statistical distribution of diameters)

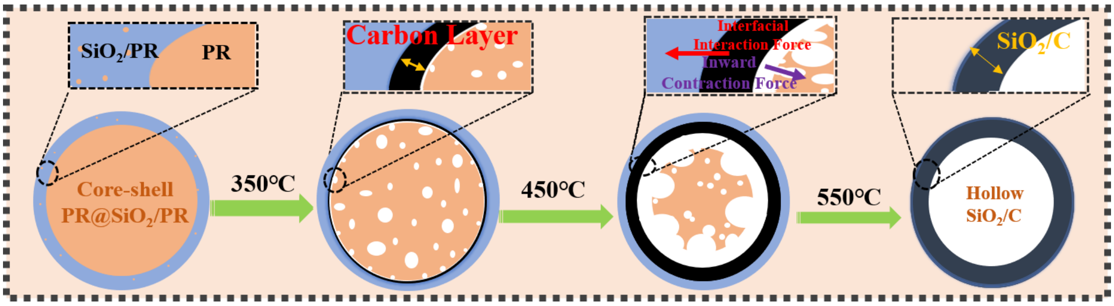

Fig. S3 The structural evolution process diagram of SiO_2/C-3 with hollow architecture

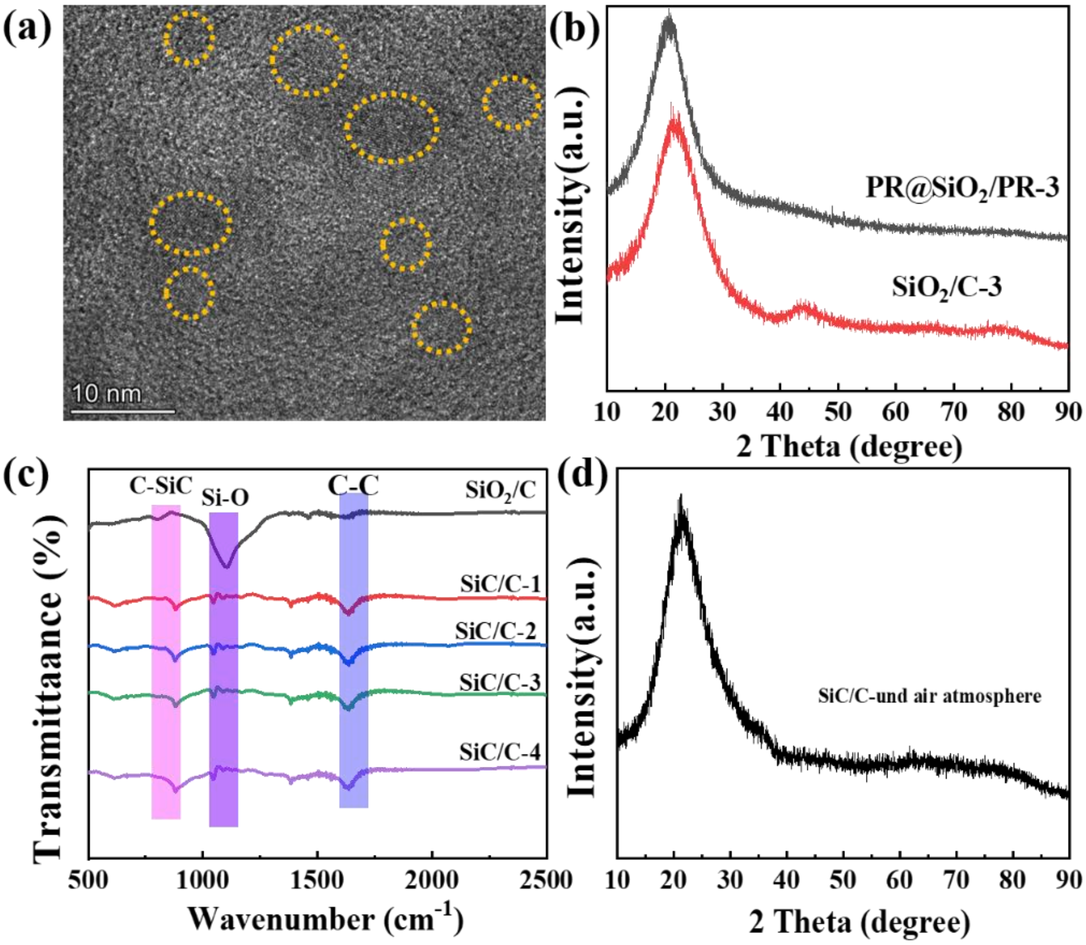

Fig. S4 **a** HR-TEM image of SiC/C-3. **b** XRD patterns of PR@SiO_2/PR-3 and SiO_2/C-3. **c** FT-IR spectra of SiO_2/C and SiC/C composites. **d** XRD patterns of SiC/C-3 after high temperature pyrolysis in air atmosphere

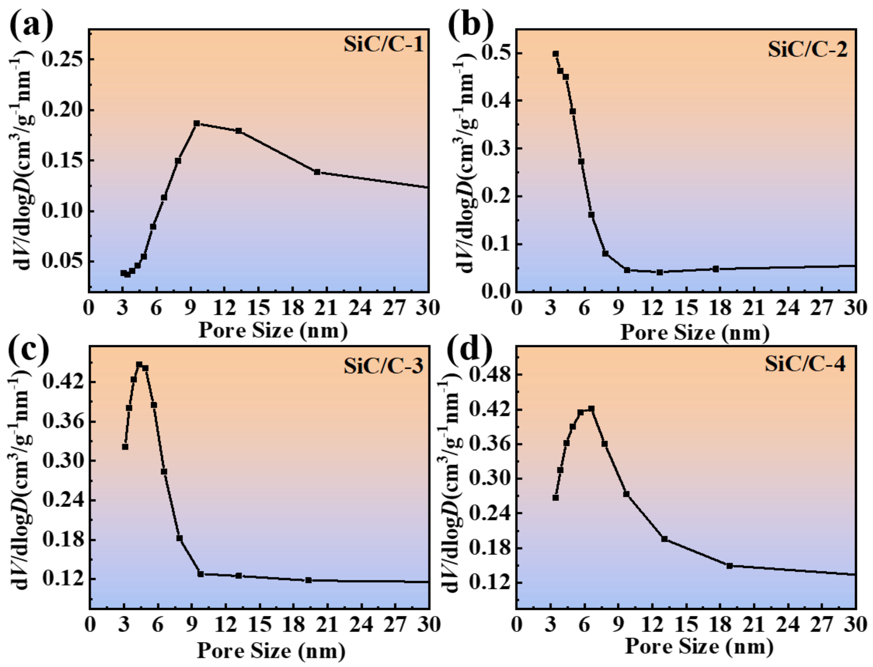

Fig. S5 Pore-size distribution of **a** SiC/C-1, **b** SiC/C-2, **c** SiC/C-3, and **d** SiC/C-4

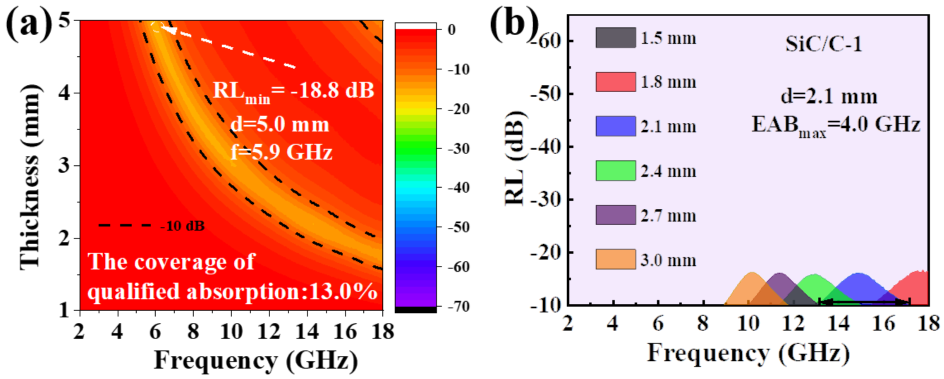

Fig. S6 a 2D RL mapping and **b** RL curves below -10 dB at selected absorber thickness of SiC/C-1

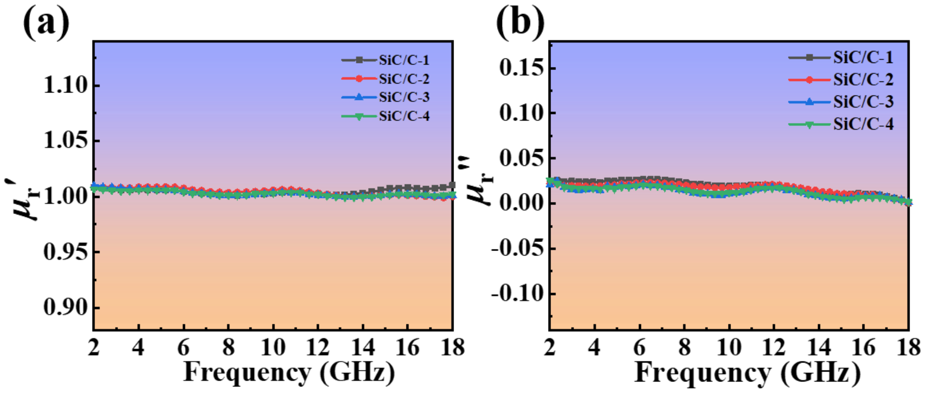

Fig. S7 Frequency-dependent **a** μ_r' curves, **b** μ_r'' curves of the relative complex permeability of SiC/C composites in the frequency range of 2.0-18.0 GHz

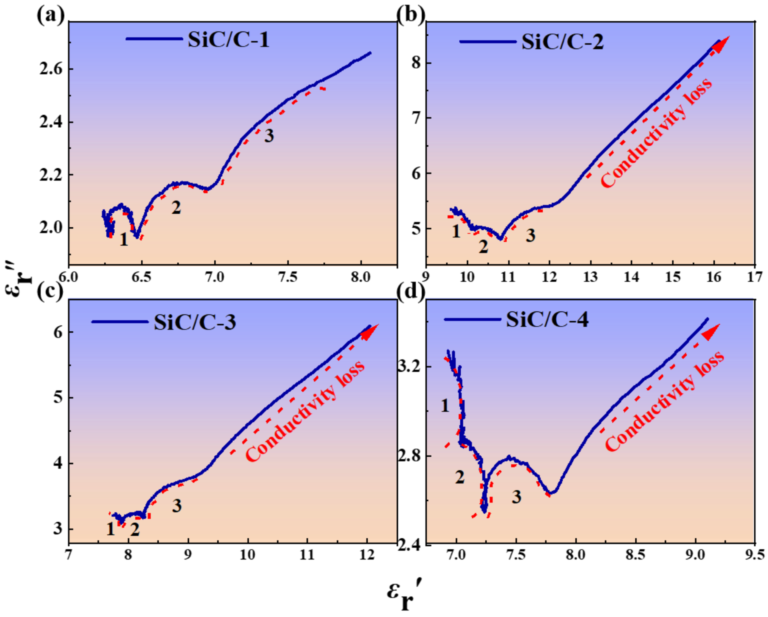

Fig. S8 Cole-Cole semicircles of **a** SiC/C-1, **b** SiC/C-2, **c** SiC/C-3, and **d** SiC/C-4

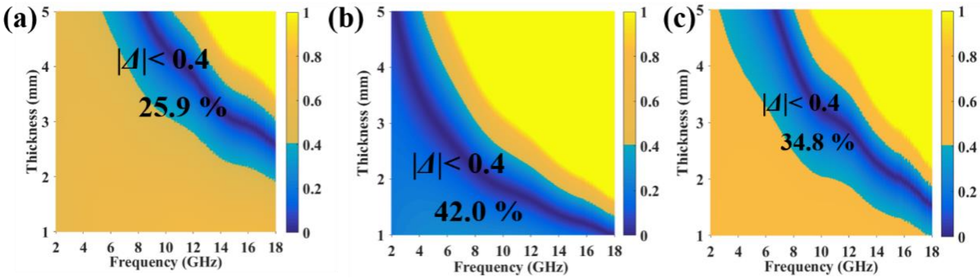

Fig. S9 A delta value (|Δ|) of **a** SiC/C-1, **b** SiC/C-2 and **c** SiC/C-4

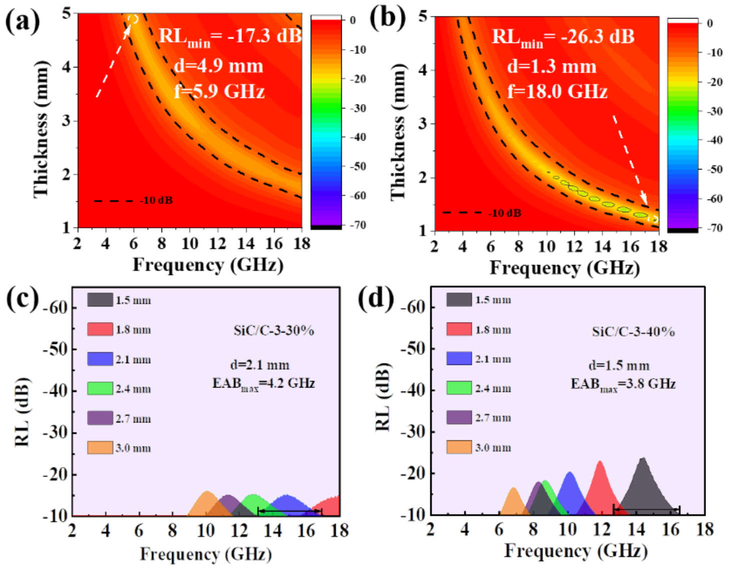

Fig. S10 2D RL mapping of **a** SiC/C-3-30% and **b** SiC/C-3-40% and RL curves below -10 dB at selected absorber thickness of **c** SiC/C-3-30% and **d** SiC/C-3-40%

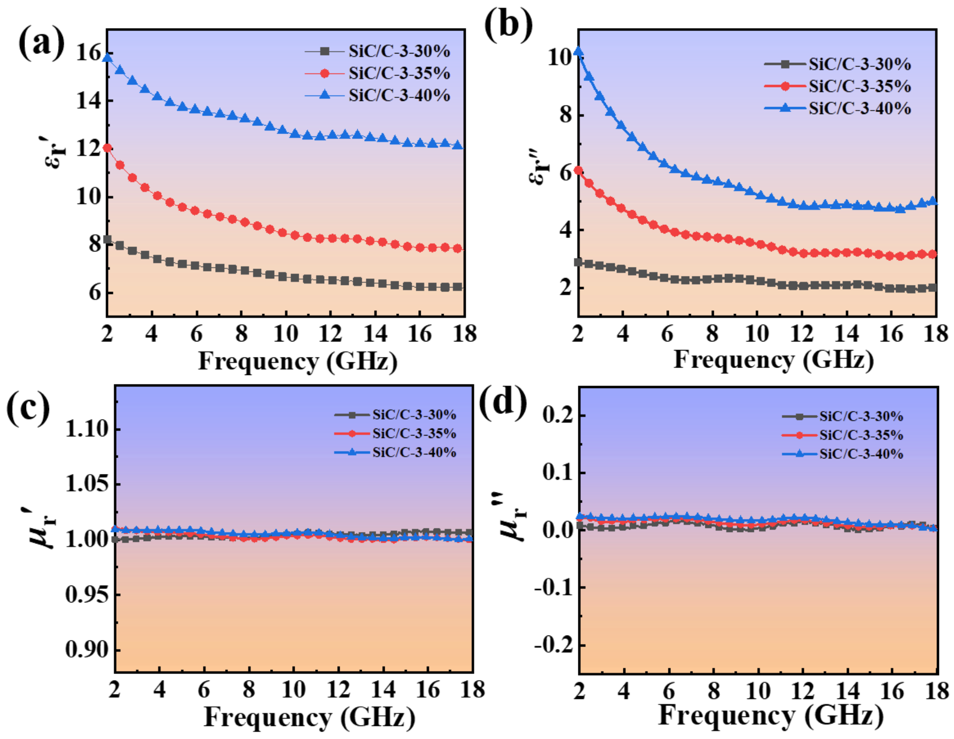

Fig. S11 Frequency-dependent **a** ε_r' curves, **b** ε_r'' curves, **c** μ_r' curves, **d** μ_r'' curves of SiC/C-3 with different filler loadings (30%, 35% and 40%)

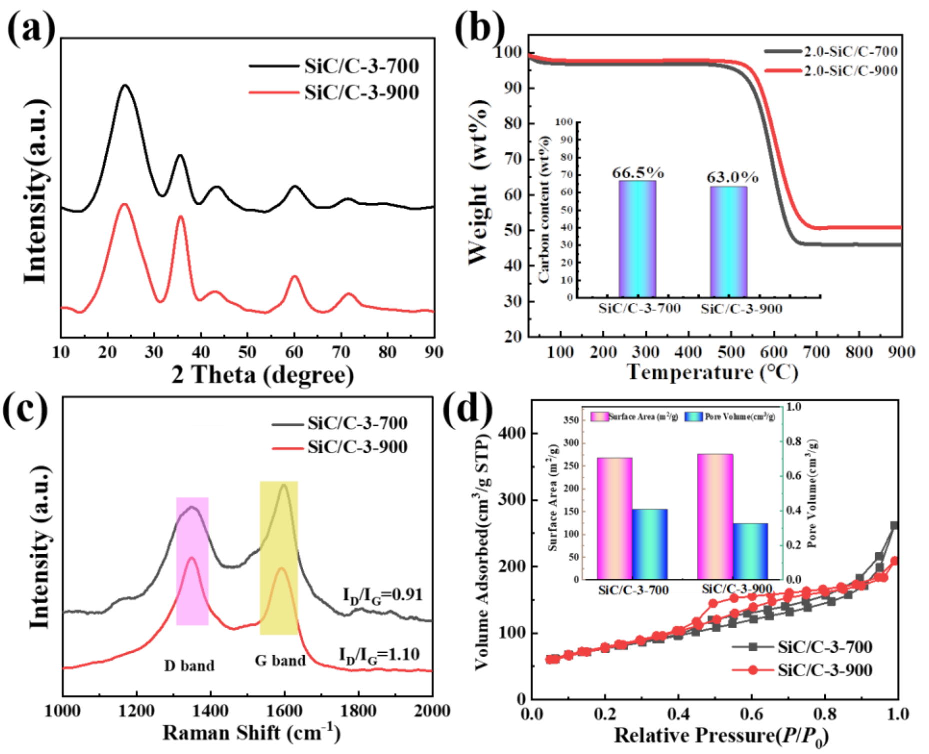

Fig. S12 a XRD pattern, **b** TG Curves (the insets are the carbon content), **c** Raman spectra and **d** N_2 adsorption/desorption isotherms (the insets are the specific surface area and total pore volume) of SiC/C-3-700 and SiC/C-3-900

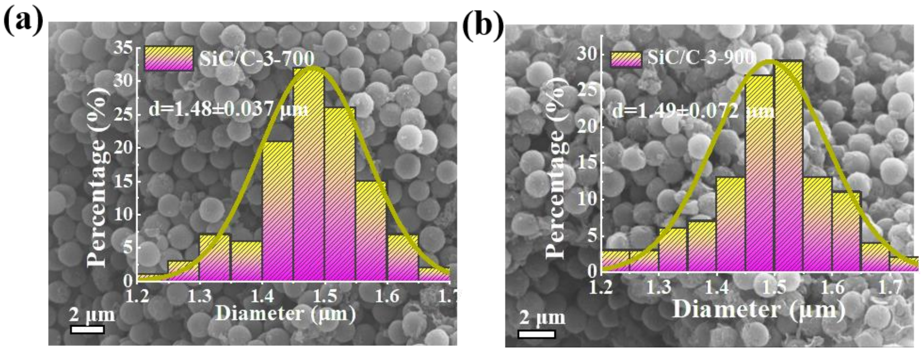

Fig. S13 SEM images of **a** SiC/C-3-700 and **b** SiC/C-3-900 (The insets are the corresponding statistical distribution of diameters)

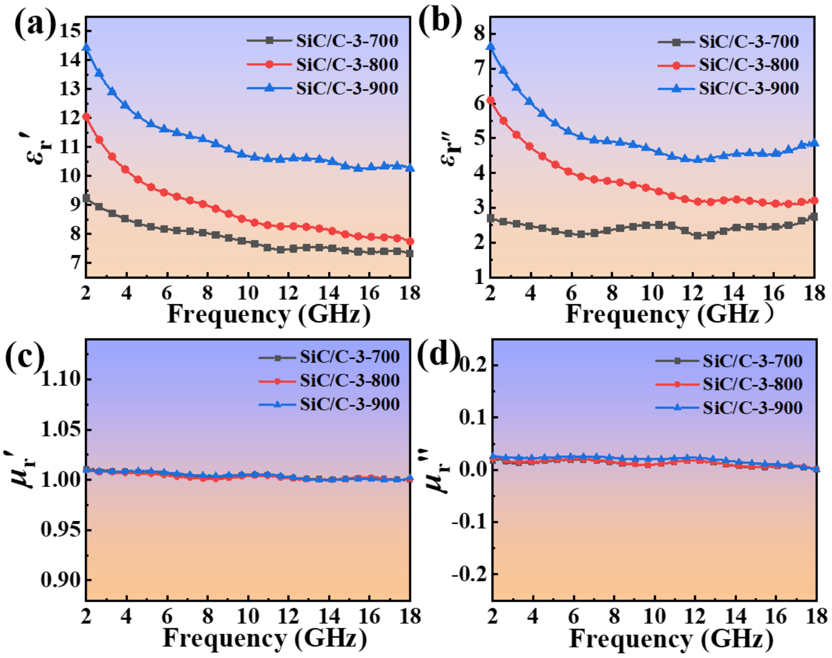

Fig. S14 Frequency-dependent **a** ε_r' curves, **b** ε_r'' curves, **c** μ_r' curves, **d** μ_r'' curves of SiC/C-3 with different pyrolysis temperature

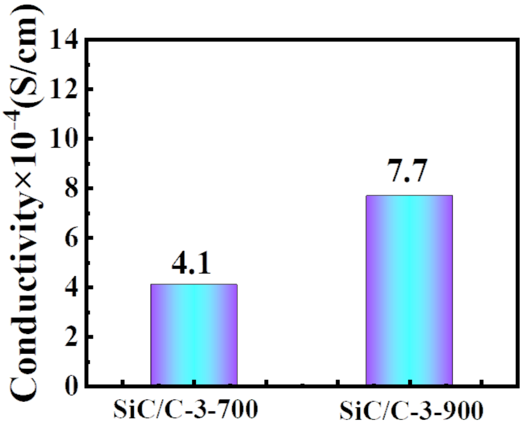

Fig. S15 The conductivity values of SiC/C-3-700 and SiC/C-3-900

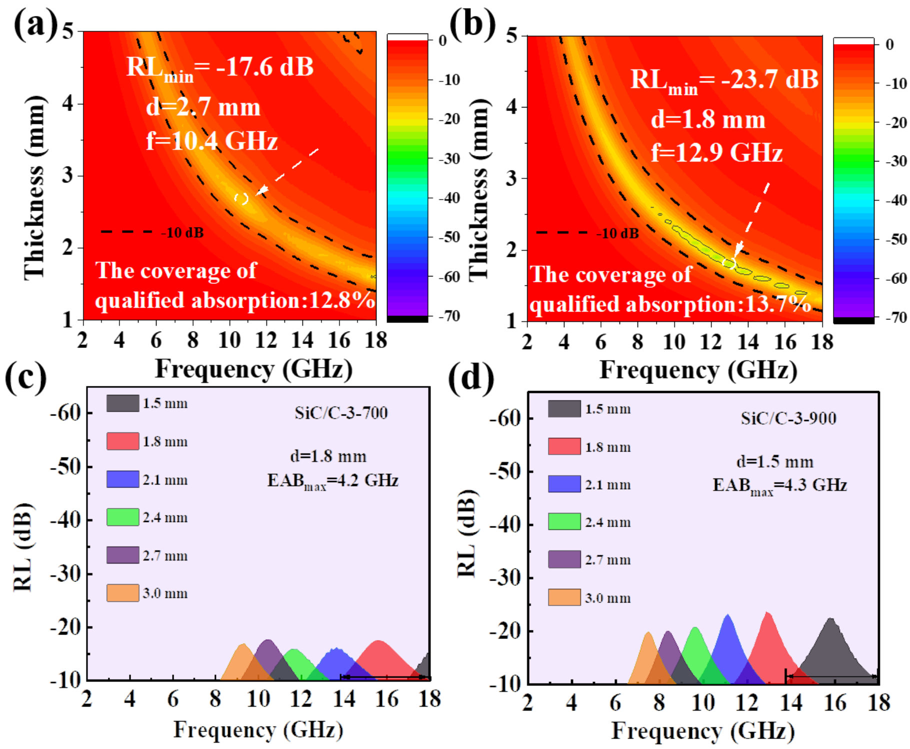

Fig. S16 2D RL mapping of **a** SiC/C-3-700 and **b** SiC/C-3-900 and **c** RL curves below -10 dB at selected absorber thickness of SiC/C-3-700 and **d** SiC/C-3-900

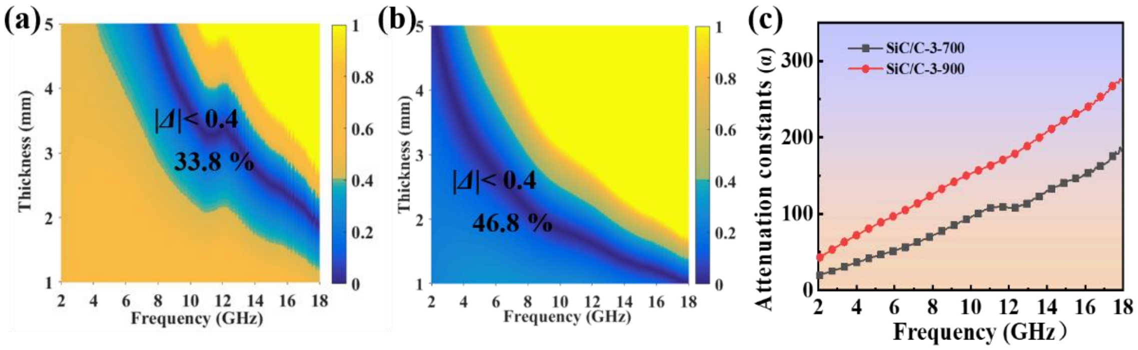

Fig. S17 a-b A delta value ($|\Delta|$) and **c** attenuation constant (α) of SiC/C-3-700 and SiC/C-3-900

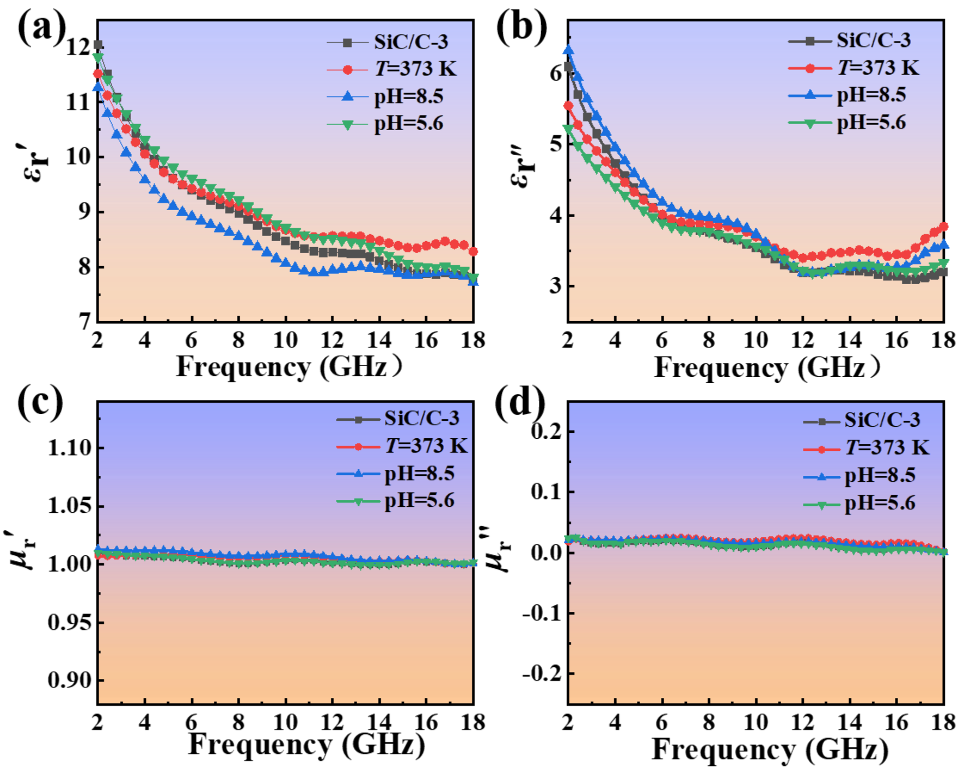

Fig. S18 Frequency-dependent **a** ε_r' curves, **b** ε_r'' curves, **c** μ_r' curves, **d** μ_r'' curves of SiC/C-3 before and after the treatments in T=373 K air atmosphere, pH=8.5 and pH=5.6 solution for 120 h, respectively

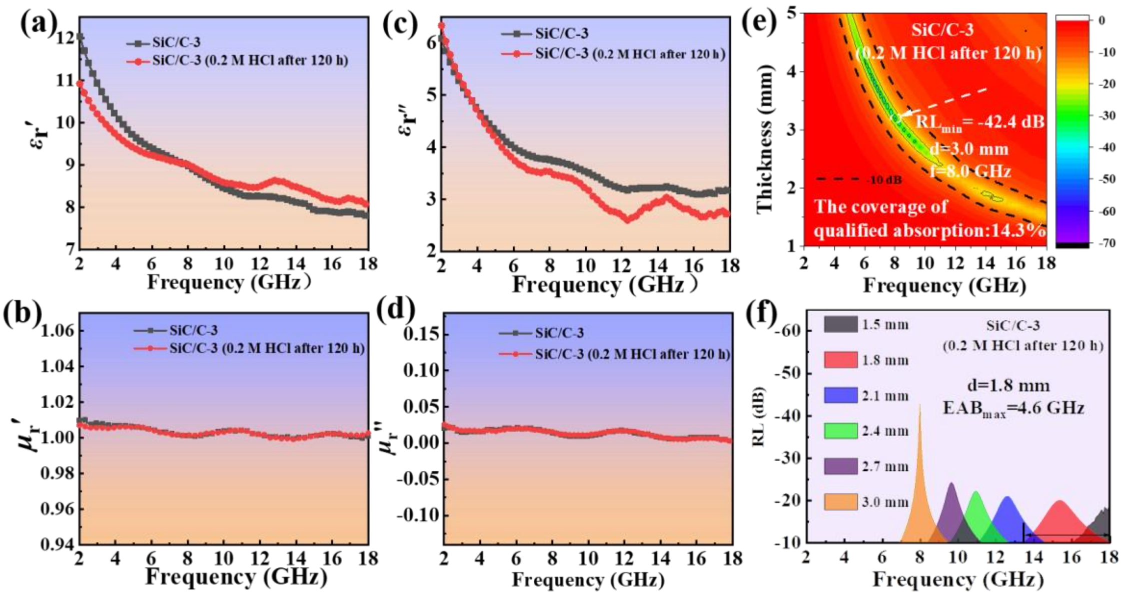

Fig. S19 Frequency-dependent **a** ε_r' curves, **b** μ_r' curves, **c** ε_r'' curves, **d** μ_r'' curves, **e** 2D RL mapping and **f** RL curves below -10 dB at selected absorber thickness of SiC/C-3 after the treatments in acid solution (0.2 M HCl after 120 h)

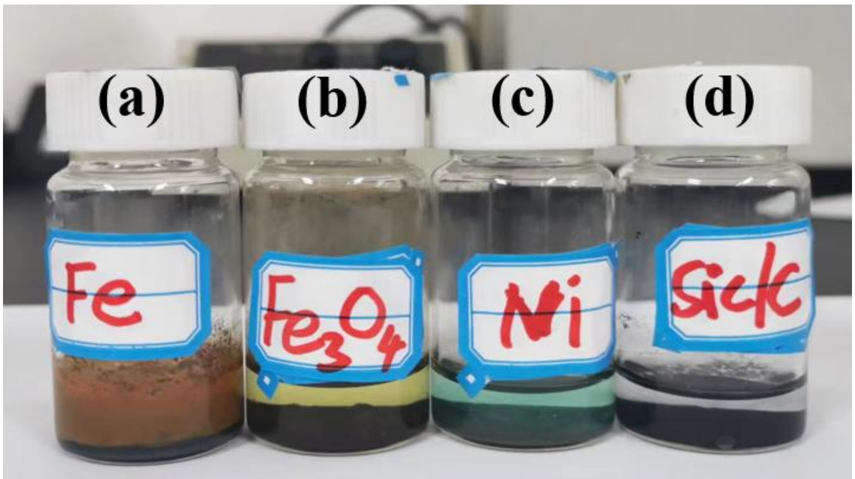

Fig. S20 Photographs of **a** Fe, **b** Fe_3O_4, **c** Ni, and **d** SiC/C-3 treated in acid solution (HCl, 0.2 M) for 120 h, respectively

Table S1 EAB, RL values, and the corresponding thicknesses of some reported MAMs composites that possess similar chemical composition with SiC/C

Samples	d (mm)	EAB (GHz)	d (mm)	RL (dB)	Refs.
SiC@C[1]	2.1	5.6	2.8	-55.4	[7]
SiC/C[2]	1.7	4.0	1.7	-43.3	[20]
rGO/SiCNW[3]	3	4.2	3	-19.6	[38]
GO@SiC[4]	3.0	4.7	3.0	-47.3	[39]
SiCnw/CF[5]	1.8	5.5	1.8	-36.5	[40]
SiC NWs/C[6]	3.3	1.2	3.3	-31.0	[41]
CNTs/SiC[7]	2	5.1	1.9	-37.6	[42]
SiC/MDCF[8]	3.5	1.6	3.6	-18.4	[43]
ZrC-SiC-C[9]	1.7	4.0	1.7	-34.8	[44]
SiC/C Foam[10]	1.4	3.8	1.4	-49.2	[45]
RGO/SiC/CNT[11]	2.0	4.2	2.9	-20.0	[46]
SWCNT/SiC[12]	2.0	4.4	2.0	-37.1	[47]
SiCnw/Cf[13]	2.0	2.4	2.0	-21.5	[48]
3D SiC/MDCF[14]	1.7	3.1	1.8	-29.5	[49]
SiC/PCF[15]	1.2	3.2	1.1	-25.9	[50]
G/SiC Fs[16]	1.4	4.4	2.1	-54.9	[51]
SiC-CNTs[17]	3.5	2.9	3.5	-61.0	[52]
SiC/C	1.8	5.1	2.7	-60.8	This work

[1]Chem. Eng. J. 433, 134484 (2022), [2]Carbon 215, 118391 (2023), [3]ACS Appl. Mater. Interfaces 9 (13), 11803-11810 (2017), [4]Chem. Eng. J. 337, 522-531 (2018), [5] Adv. Eng. Mater. 23 (10), 2100434 (2021), [6]Carbon 122, 718-725 (2017), [7]J. Alloys Compd. 520, 295-300 (2012), [8]ACS Sustainable Chem. Eng. 7 (2), 2774-2783 (2019), [9]Appl. Surf. Sci. 591, 153105 (2022), [10]J. Alloys Compd. 879, 160440 (2021), [11]Ceram. Int. 46 (11), 18160-18167 (2020), [12]J. Electron. Mater. 49 (12), 7279-7291 (2020), [13]J. Alloys Compd. 687, 833-838 (2016), [14]J. Adv. Ceram. 8 (4), 479-488 (2019), [15]J. Alloys Compd. 791, 883-891 (2019), 16, [16]Carbon 182, 435-444 (2021), [17]Ceram. Int. 47 (6), 8123-8132 (2021).
